# Dose-Related Associations of a Palm Kernel Meal-Derived Mannan-Oligosaccharide-Containing Hydrolysate with Selected Immune and Ruminal Fermentation Measures in Growing Goats

**DOI:** 10.3390/vetsci13101008

**Published:** 2026-09-24

**Authors:** Natthrit Roekngam, Supinya Chuchai, Porntip Thongmanee, Niramol Thongtem, Suwichak Chaisit, Wiruntita Bohman, Sunisa Khongthong

**Affiliations:** 1 Faculty of Veterinary Science, Rajamangala University of Technology Srivijaya, Nakhon Si Thammarat 80240, Thailand; natthrit.r@rmutsv.ac.th (N.R.); supinya.c@rmutsv.ac.th (S.C.); porntip.t@rmutsv.ac.th (P.T.); niramol.t@rmutsv.ac.th (N.T.); wiruntita.c@rmutsv.ac.th (W.B.); 2 Department of Chemical Engineering and Pharmaceutical Chemistry, School of Engineering and Technology, Walailak University, Nakhon Si Thammarat 80160, Thailand; suwichak.ch@mail.wu.ac.th; 3 Biomass and Oil Palm Center of Excellence, Walailak University, Nakhon Si Thammarat 80160, Thailand

**Keywords:** agro-industrial by-product, cytokines, immunoglobulin A, inflammatory signaling, prebiotic, small ruminants, volatile fatty acids, circular bioeconomy

## Abstract

Mannan-oligosaccharides (MOS) may influence microbial and immune-related processes, but evidence concerning palm kernel meal (PKM)-derived MOS-containing products in goats remains limited. The experimental product was a chemically heterogeneous PKM-derived hydrolysate in which M2–M6 oligosaccharides were identified by HPLC–RID; residual sugars and other soluble constituents were also present. Twenty growing male goats received 0, 2.5, 5.0, or 10.0 g hydrolysate/goat/day (*n* = 5 per treatment) for 90 days. Feed intake, growth, hematological and serum biochemical indices, circulating immune indicators, peripheral blood leukocyte gene expression, and selected ruminal measures were evaluated. Immune and gene-expression outcomes were measured in prefeeding blood collected on Day 90. Ruminal pH and acetate, propionate, and butyrate were measured in a single rumen-fluid sample collected 3 h after feeding on Day 90. Serum IgA showed a positive linear association with supplementation level, whereas IL-1β and IL-6 showed negative associations; these remained significant after false-discovery-rate correction (*q* = 0.036, 0.012, and 0.012, respectively). Selected immune-related transcripts also differed among treatments, but these endpoint patterns do not establish coordinated immunological regulation or a specific mechanism. Propionate and butyrate showed nominal positive linear associations, whereas the acetate-to-propionate ratio showed a nominal negative association (raw *p* = 0.028, 0.021, and 0.018, respectively); none remained significant after correction (*q* = 0.056 for each). Ruminal pH, the sum of the three measured VFA concentrations, feed intake, and growth did not differ detectably. No overt clinical abnormalities requiring veterinary intervention were observed, but the study was not powered to establish safety. These preliminary between-group endpoint associations concern the complete heterogeneous hydrolysate and cannot be attributed specifically to M2–M6 oligosaccharides. Larger, adequately powered studies with pretreatment and repeated measurements are required.

## Simple Summary

Palm kernel meal contains mannose-rich carbohydrates that can be hydrolyzed to produce mannan-oligosaccharides. In this study, palm kernel meal was processed into a chemically heterogeneous hydrolysate containing identified mannan-oligosaccharides and other soluble components. Twenty growing male goats received 0, 2.5, 5.0, or 10.0 g of the hydrolysate per animal daily for 90 days. Feed intake and growth performance did not differ detectably among treatments. At the Day 90 endpoint, increasing supplementation levels were associated with higher serum immunoglobulin A and lower interleukin-1β and interleukin-6 concentrations, together with differences in selected immune-related transcripts. These immune associations remained significant after correction for multiple testing. Propionate, butyrate, and the acetate-to-propionate ratio also showed dose-related patterns in a single rumen-fluid sample, but these associations did not remain significant after multiple-testing correction. Pretreatment immune and ruminal measurements were unavailable, and only five goats were included per treatment. Therefore, the findings are preliminary and do not establish improved immune function, sustained changes in ruminal fermentation, safety, or a recommended dose. Because the hydrolysate contained multiple components, the observed associations cannot be attributed specifically to mannan-oligosaccharides. Larger, adequately powered studies with repeated measurements and further product characterization are required.

## 1. Introduction

Growing goats are exposed to nutritional, management, and environmental factors that can influence gastrointestinal function, immune status, and productive performance. In ruminants, microbial fermentation supplies volatile fatty acids (VFAs) that contribute substantially to host energy metabolism, while the gastrointestinal tract also forms an important interface among dietary components, microorganisms, and the immune system. Mannan-oligosaccharides (MOS) have therefore been investigated as feed ingredients with potential effects on gastrointestinal and immune-related outcomes. However, responses reported in goats and sheep have varied among studies and outcomes [1,2,3].

Several mechanisms have been proposed for MOS-containing products, but their relevance is context-dependent. Mannose-containing structures may bind mannose-specific type 1 fimbriae, including FimH-containing adhesins expressed by some enteric bacteria, and thereby interfere with bacterial attachment to epithelial surfaces [4]. FimH can also be recognized through TLR4-dependent innate signaling in experimental systems [5]. These observations do not establish that dietary MOS directly regulates TLR4/NF-κB signaling in growing goats. The present study did not evaluate FimH-expressing bacteria, bacterial adhesion, pathogen abundance, or TLR4/NF-κB protein activation; consequently, these pathways provide biological context rather than mechanisms tested in this experiment.

MOS-containing supplements have also been associated with differences in nutrient utilization, ruminal nitrogen metabolism, and fermentation measures, although results in small ruminants have been inconsistent [2,3]. Such variation may reflect differences in MOS source and chemical composition, basal diet, dose, animal age and physiological status, experimental duration, and sampling time. Potential effects on ruminal fermentation are also distinct from FimH-mediated interference with enteric bacterial adhesion and should not be presented as a single established pathway connecting dietary MOS with systemic immune responses.

Palm kernel meal (PKM) and palm kernel cake (PKC) are co-products of palm kernel oil production and contain substantial amounts of mannose-rich cell-wall polysaccharides. Structural analysis indicated that minimally substituted linear mannans represented approximately 78% of the non-starch-polysaccharide fraction of palm-kernel meal [6]. In a separate PKC study, total carbohydrate constituted 60.3% (*w*/*w*) of the material, and a previously reported estimate placed mannan at approximately 57.8% of the hemicellulose fraction [7]. These values were calculated on different analytical bases and should not be interpreted as directly comparable estimates of whole-material mannan content. Composition may also vary with palm source, oil-extraction method, residual oil, shell contamination, particle size, and analytical procedure. The initial polymeric mannan concentration of the PKM batch used in the present study was not directly quantified.

The mannose-rich polysaccharide fraction nevertheless makes PKM a potential substrate for producing manno-oligosaccharide-containing hydrolysates. Oligosaccharides have been extracted from PKC under defined processing conditions [8], and enzymatic hydrolysis studies have demonstrated the release of mannose and oligosaccharides with different degrees of polymerization [7,9,10]. Product recovery and composition depend on the starting material, pretreatment, enzyme specificity and activity, solids loading, hydrolysis conditions, and downstream separation. Accordingly, the experimental product is described here as a chemically heterogeneous PKM-derived MOS-containing hydrolysate in which M2–M6 oligosaccharides were identified by HPLC–RID. Because the HPLC–RID procedure was used for identification rather than validated quantification, and because residual sugars and other soluble constituents were present, the hydrolysate should not be considered purified or quantitatively defined MOS.

Evidence from ruminants remains outcome-specific. In sheep, MOS supplementation has been associated with greater apparent digestibility of selected nutrients, nitrogen retention, and some antioxidant indices, but these findings were not accompanied by consistent improvements in feed intake or growth [2]. In growing rams, graded MOS supplementation altered selected digestibility and ruminal nitrogen measures but did not detectably affect feed intake, body weight gain, hematological variables, or the measured ruminal short-chain fatty-acid profile [3]. In neonatal goats, MOS supplementation was associated with differences in cytokine concentrations, ileal secretory IgA, intestinal immune-related gene expression, and microbial composition [1]. Extrapolation of these findings to growing goats is limited by differences in age, digestive development, diet, product composition, experimental conditions, and statistical power.

Evidence concerning graded supplementation with a PKM-derived MOS-containing hydrolysate in growing goats is therefore limited. The novelty of the present study lies in the concurrent evaluation of selected circulating immune indicators, peripheral blood leukocyte gene expression, and endpoint ruminal fermentation measures across four supplementation levels, together with feed intake, growth performance, and selected hematological and serum biochemical indices. This design permits exploratory characterization of dose-response patterns, but it cannot attribute any observed association specifically to the identified M2–M6 oligosaccharides.

The objective was to evaluate associations between graded supplementation with the complete PKM-derived hydrolysate (0, 2.5, 5.0, and 10.0 g/goat/day) and selected immune, gene-expression, and endpoint ruminal fermentation measures in growing goats. We hypothesized that increasing supplementation would be associated with higher circulating IgA, lower pro-inflammatory indicators, higher ruminal propionate, and a lower acetate-to-propionate ratio without a marked reduction in ruminal pH. Feed intake, growth performance, hematological and serum biochemical indices, and the remaining immune and fermentation measures were evaluated as supporting outcomes.

## 2. Materials and Methods

### 2.1. Source and Preparation of Palm Kernel Meal

Palm kernel meal (PKM) was obtained from Palmtongkham Company, Phra Saeng District, Surat Thani Province, Thailand, in March 2025. PKM was produced as a by-product of palm kernel oil extraction using a mechanical expeller process. A single production batch was used throughout the preparation of the MOS containing hydrolysate and the subsequent animal-feeding experiment to minimize variation in the composition of the starting material. Upon receipt, the PKM was dried overnight in a forced-air oven (model UF110; Memmert GmbH + Co., KG, Schwabach, Germany) at 60 °C. The dried material was ground using an MF 10 basic laboratory mill equipped with a 1-mm screen (IKA-Werke GmbH & Co., KG, Staufen, Germany). The prepared PKM was subsequently stored in sealed polyethylene bags at ambient laboratory temperature until hydrolysate production. All bags were labeled with the source, production-batch code, preparation date, and sample identification.

### 2.2. Production of PKM-Derived Mannan-Oligosaccharide-Rich Hydrolysate

PKM-derived MOS-containing hydrolysate was produced by alkaline pretreatment followed by enzymatic hydrolysis, with modifications to previously described methods [7,8,9,10]. No defatting step was performed. The prepared PKM was suspended in 2% (*w*/*v*) sodium hydroxide at a solid-to-liquid ratio of 1:10 (*w*/*v*) and autoclaved at 120 °C for 2 h. After cooling, the suspension was neutralized to pH 6.5–7.0 with 1 N hydrochloric acid under continuous stirring and subjected to suction filtration through Whatman No. 1 filter paper (Cytiva, Maidstone, UK). The insoluble fraction was recovered for enzymatic hydrolysis.

The pretreated solid was suspended in 50 mM sodium acetate buffer (pH 5.5) at a solid-to-liquid ratio of 1:10 (*w*/*v*). Mannanase powder (iKnowZyme Mannanase Powder; Reach Biotechnology Co., Ltd., Pathum Thani, Thailand; declared activity, 10,000 U/g) was added at 200 U/g of dry pretreated substrate, equivalent to 20 mg enzyme preparation per gram of substrate. The mixture was incubated at 50 °C for 24 h with agitation at 150 rpm. The enzyme was subsequently inactivated at 100 °C for 15 min, and the mixture was cooled and filtered through Whatman No. 1 filter paper. The soluble filtrate was collected as the crude MOS-rich hydrolysate.

The filtrate was frozen and lyophilized for approximately 4 days using a Delta 2-24 LSC freeze dryer (Martin Christ Gefriertrocknungsanlagen GmbH, Osterode am Harz, Germany) at the Office of Scientific Instrument and Testing, Prince of Songkla University, Hat Yai, Songkhla, Thailand. The resulting powder was weighed and stored in airtight containers in a desiccator at room temperature. Production was independently performed in three processing runs using separate portions of the same homogenized PKM batch. The dry hydrolysate yield was calculated relative to the initial dry weight of PKM and averaged 12.58 ± 0.42% (*w*/*w*).

### 2.3. Chemical Characterization of the Hydrolysate

#### 2.3.1. Proximate and Carbohydrate Composition

The freeze-dried hydrolysate from each of the three independent processing runs was analyzed in triplicate for moisture, ash, crude protein, total carbohydrate, reducing sugar, water solubility, and pH. Moisture was determined by drying approximately 2 g of sample at 105 °C to a constant weight using the same forced-air oven, whereas ash was determined by incineration at 550 °C for 4 h. Crude protein was measured using the Kjeldahl method with a nitrogen-to-protein conversion factor of 6.25. These analyses followed standard AOAC procedures [11].

Total carbohydrate was determined using the phenol–sulfuric acid method, with absorbance measured at 490 nm [12]. Reducing sugar was determined using the 3,5-dinitrosalicylic acid method after heating the reaction mixture for 5 min, followed by absorbance measurement at 540 nm [13]. D-Mannose was used to construct the calibration curves for both assays, and the results were expressed as D-mannose equivalents.

Water solubility was determined using a gravimetric procedure adapted from Cano-Chauca et al. [14]. One gram of hydrolysate was dispersed in 100 mL of distilled water, stirred for 30 min, and centrifuged at 3000× *g* for 10 min using an NF 1200R refrigerated centrifuge (Nüve Sanayi Malzemeleri İmalat ve Ticaret A.Ş., Ankara, Türkiye). A measured portion of the supernatant was dried at 105 °C to a constant weight, and solubility was reported as the percentage of dissolved solids relative to the initial dry sample weight. The pH was measured in a 1% (*w*/*v*) aqueous hydrolysate solution using a FiveEasy F20 digital pH meter (Mettler-Toledo GmbH, Greifensee, Switzerland) calibrated with pH 4.00 and 7.00 buffers. Moisture content was expressed on an as-is basis. Total carbohydrate, reducing sugar, crude protein, ash, and the fraction calculated by difference were expressed on a dry matter basis. Water solubility was calculated relative to the initial dry sample weight. Because reducing sugars were included within the total-carbohydrate measurement, they were not treated as a separate component when calculating the unaccounted fraction. The unaccounted fraction was calculated on a dry matter basis by subtracting the percentages of ash, crude protein, and total carbohydrate from 100. Results from the three technical determinations were averaged within each processing run, and the values from the three independent processing runs were subsequently reported as mean ± SD.

#### 2.3.2. HPLC–RID Identification and Quantification of Manno-Oligosaccharides

The freeze-dried hydrolysates were analyzed by high-performance liquid chromatography with refractive-index detection (HPLC–RID), using an approach adapted from previously reported analyses of PKM- or PKC-derived sugars and manno-oligosaccharides [7,8,9,10]. The analysis was used to identify D-mannose and manno-oligosaccharides with degrees of polymerization of two to six by retention-time matching with authentic standards. The method was not validated for quantitative determination.

Hydrolysate from each processing run was dissolved separately in HPLC-grade water at 10 mg/mL, centrifuged at 10,000× *g* for 10 min, and filtered through a 0.22-µm nylon membrane. The filtrate was diluted fivefold with HPLC-grade water, maintained at 4 °C, and analyzed within 24 h.

Chromatographic analysis was performed using an LC-20AD system equipped with an RID-20A refractive-index detector (Shimadzu Corporation, Kyoto, Japan) and an Inertsil NH_2_ column (250 × 4.6 mm, 5 µm; GL Sciences Inc., Tokyo, Japan) maintained at 35 °C. Acetonitrile–water (70:30, *v*/*v*) was delivered isocratically at a flow rate of 1.0 mL/min. The injection volume was 20 µL, and the total run time was 30 min.

Authentic D-mannose, mannobiose (M2), mannotriose (M3), mannotetraose (M4), mannopentaose (M5), and mannohexaose (M6) standards (Megazyme/Neogen, Bray, Ireland) were analyzed under the same chromatographic conditions to establish their retention times. Sample peaks were assigned to the corresponding standards when the difference in retention time was no greater than ±2%. Hydrolysates from the three independent processing runs were prepared separately, and each preparation was injected in triplicate. Technical injections were used to evaluate consistency of the retention-time assignments.

Because the procedure was not subjected to full analytical validation and the peak identities were not confirmed using an orthogonal technique such as mass spectrometry, the chromatographic results were interpreted as retention-time-based identification of M2–M6 rather than validated quantitative measurements. Consequently, concentrations in mg/g, percentage composition, and total M2–M6 content were not calculated from these chromatograms.

### 2.4. Animals, Ethics, and Experimental Location

The animal-feeding experiment was conducted at Phakphum Farm, Khlong Thom District, Krabi Province, Thailand. Twenty intact male growing crossbred meat goats (*Capra hircus*), with a mean age of approximately 4 months and 20 days and an overall initial body weight of 17.50 ± 3.10 kg (mean ± SD; range, 12.9–23.0 kg), were enrolled.

Eligibility was assessed before treatment allocation. Each goat underwent a physical examination by the project veterinarian, including assessment of general appearance, body condition, behavior, appetite, rectal temperature, heart and respiratory rates, mucous membranes, ruminal motility, locomotion, and fecal consistency. Goats were eligible when they were 4–5 months old, weighed 12–25 kg, exhibited normal appetite and activity, had normal fecal consistency, and showed no clinically apparent infectious, gastrointestinal, respiratory, or musculoskeletal disease. Exclusion criteria included congenital abnormalities, persistent diarrhea, respiratory signs, moderate-to-severe lameness, systemic illness, or antimicrobial treatment within the 30 days preceding enrollment.

Before enrollment, all goats underwent the farm’s routine preventive-health program under veterinary supervision, including vaccination against clostridial diseases, anthelmintic treatment, and vitamin A, D_3_, and E supplementation. Product identities, doses, routes of administration, and treatment dates were obtained from the farm health records. Only goats considered clinically healthy following completion of the preventive-health procedures were enrolled.

Prespecified withdrawal criteria included persistent anorexia, persistent diarrhea, respiratory disease, moderate-to-severe lameness, systemic illness, or any condition requiring therapeutic intervention that could interfere with the study outcomes. Any goat meeting a withdrawal criterion was to be removed from the experiment and receive appropriate treatment under veterinary supervision.

The study protocol was reviewed and approved by the Institutional Animal Care and Use Committee of Rajamangala University of Technology Srivijaya, Thailand (protocol no. IAC 05-04-2025; approved 15 July 2025). Animal care and experimental procedures were conducted in accordance with the Ethical Principles and Guidelines for the Procedures on Animals for Scientific Purposes of the National Research Council of Thailand. The study is reported in accordance with the ARRIVE 2.0 guidelines [15].

Animal welfare was assessed by trained personnel at least twice daily. Observations included general appearance, behavior, appetite, feed and water consumption, fecal consistency, locomotion, respiratory signs, and observable evidence of pain or systemic illness. Clinical abnormalities, veterinary assessments, treatments, and withdrawal decisions, if any, were recorded throughout the experiment.

### 2.5. Experimental Design and Dietary Treatments

Following a 14-day adaptation period, the 20 goats were allocated to four dietary treatments in a randomized complete block design, with five goats per treatment. Goats were ranked from the lowest to the highest initial body weight and sequentially arranged into five blocks of four goats with similar body weights. Within each block, one goat was randomly assigned to each of the four treatments by drawing lots, ensuring that every treatment was represented once within each block. Each goat was housed and fed individually and was considered one experimental unit. The experimental feeding period lasted 90 days.

No formal a priori sample-size calculation was performed specifically for this experiment, and no single primary outcome was used to determine the sample size. The number of animals was based on the availability of eligible goats and the feasibility of implementing a randomized complete block design comprising five initial-body-weight blocks, with all four treatments represented once within each block, while limiting animal use. Accordingly, the experiment was designed as a preliminary dose-response study, and the resulting estimates should be interpreted in light of the limited sample size.

The four treatments consisted of the basal diet without the PKM-derived MOS-containing hydrolysate (CON; 0 g/goat/day) or the same basal diet supplemented with the hydrolysate at 2.5 g/goat/day (MOS2.5), 5.0 g/goat/day (MOS5), or 10.0 g/goat/day (MOS10). Because no established effective dose of this PKM-derived hydrolysate was available for growing goats, the supplementation levels were selected as exploratory graded doses representing low, intermediate, and high daily amounts that could be administered individually. The selected levels were intended to characterize potential dose-response patterns across the tested range rather than to determine an optimal or recommended practical dose.

Fixed daily amounts were administered on a per-animal basis and were not adjusted as body weight increased during the 90-day feeding period. Consequently, the amount supplied per kilogram of body weight decreased as the goats grew. Dose-response analyses were therefore based on the assigned supplementation levels of 0, 2.5, 5.0, and 10.0 g/goat/day and should not be interpreted as responses to constant body-weight-normalized doses.

The assigned amount of hydrolysate was weighed separately for each goat each day. No carrier was used; therefore, carrier compensation was not required for the CON group. To promote complete consumption, the hydrolysate was mixed with a small portion of the basal diet and offered individually before the remainder of the daily ration, as described in Section 2.6.

The study was conducted as an open-label experiment. Blinding was not implemented because the same research team was responsible for preparing and administering the treatment-specific supplements, monitoring the animals, collecting and processing samples, conducting laboratory analyses, and managing and analyzing the dataset. Operational separation of these activities was not feasible within the personnel and resources available for this preliminary experiment. Consequently, personnel involved in animal care, sample collection, laboratory analyses, and statistical analysis were aware of the treatment assignments.

To reduce procedural and analytical variation, animal management, feeding procedures, sampling times, sample-collection procedures, sample-processing and storage conditions, assay protocols, and instrument settings were standardized across treatment groups. Laboratory outcomes were obtained using instrument-based assays and prespecified calculation procedures, and samples were analyzed in duplicate where indicated in the relevant Methods sections. The same outcome definitions and statistical models were applied to all treatment groups. These measures did not eliminate the possibility of detection or analytical bias arising from the absence of blinding, which is acknowledged as a study limitation. The randomization procedure, experimental unit, sample-size determination, and absence of blinding are reported in accordance with the ARRIVE 2.0 guidelines [15].

### 2.6. Housing, Diet, and Feeding Management

Goats were housed individually in an open-sided, naturally ventilated barn raised above ground level. Each goat was maintained in a 1.2 × 0.8 m pen with wooden slatted flooring and was provided with an individual feed trough and water container. Clean drinking water and mineral salt blocks were available *ad libitum* throughout the adaptation and experimental periods. Pens, feed troughs, and water containers were cleaned routinely. The goats retained visual and auditory contact with adjacent animals; no additional environmental-enrichment program was implemented. Animal health and welfare monitoring was performed as described in Section 2.4.

All goats received the same basal total mixed ration (TMR), consisting of concentrate and Pangola grass hay at a ratio of 70:30 on a dry matter basis. The diet was formulated to meet or exceed the nutrient requirements of growing goats with a target average daily gain of approximately 200 g/day, according to the recommendations of the National Research Council [16]. The ingredient composition and calculated chemical composition of the basal TMR are presented in Table 1.

The TMR was offered *ad libitum* twice daily at 07:00 and 16:00 h. The amount offered was adjusted daily to maintain approximately 10% refusals. For all goats, including those in the CON group, approximately 200 g of the basal TMR were withheld from the morning ration and offered first as the treatment-delivery portion. The assigned amount of PKM-derived MOS-containing hydrolysate was weighed separately for each goat, thoroughly hand-mixed with this portion, and offered individually before the remainder of the morning ration. The treatment-delivery portion was therefore part of, rather than additional to, the daily TMR allowance.

Consumption of the treatment-delivery portion was monitored daily by direct observation and inspection of the feed trough. All goats completely consumed this portion before the remainder of the morning ration was offered. Consequently, actual hydrolysate intake corresponded to the assigned doses of 0, 2.5, 5.0, and 10.0 g/goat/day, and no correction for hydrolysate remaining in feed refusals was required. The dry matter supplied by the consumed hydrolysate was included in the calculation of total dry matter intake, as described in Section 2.7.

### 2.7. Feed Sampling and Chemical Analysis

The amounts of total mixed ration (TMR) offered and refused were weighed and recorded daily for each goat. Basal TMR dry matter intake was calculated as the dry matter offered minus the dry matter refused, using the dry matter concentrations of the corresponding feed and refusal samples. The dry matter contributed by the consumed hydrolysate was then added to the basal TMR dry matter intake to calculate total dry matter intake (DMI). Thus, the reported DMI included dry matter from both the basal TMR and the supplemented hydrolysate. As described in Section 2.6, the treatment-containing portion was completely consumed before the remainder of the ration was offered; therefore, the refusals contained no hydrolysate, and the actual hydrolysate intake corresponded to the assigned dose.

Representative samples of the offered TMR and individual refusals were collected weekly. Samples were stored at −20 °C and composited by animal within each 30-day period in proportion to the amount collected. Subsamples were dried in a forced-air oven at 60 °C for 48 h in the same forced-air oven and subsequently ground through a 1-mm screen using the same laboratory mill, and stored in sealed containers until chemical analysis.

Dry matter (DM; method 934.01), ash (method 942.05), crude protein (CP; method 984.13), and ether extract (EE; method 920.39) were determined according to AOAC procedures [11]. Neutral detergent fiber (NDF) was determined using heat-stable α-amylase and sodium sulfite, and acid detergent fiber (ADF) was determined according to the detergent-fiber procedures described by Van Soest et al. [17]. Neutral detergent fiber and ADF were expressed exclusive of residual ash. Acid detergent lignin was determined by treating the acid-detergent residue with 72% sulfuric acid. All chemical analyses were performed in duplicate for each composite sample.

Non-fibrous carbohydrate (NFC) was calculated by difference as 100 minus the sum of CP, NDF, EE, and ash, with all components expressed as percentages of DM. Total digestible nutrients (TDN) were estimated using the summative approach of Weiss et al. [18], based on the predicted digestible fractions of NFC, CP, fatty acids, and NDF. Digestible energy (DE; Mcal/kg DM) was estimated by multiplying TDN, expressed as a percentage of DM, by 0.04409. Metabolizable energy (ME; Mcal/kg DM) was estimated as 0.82 × DE, following the energy-conversion approach described for small-ruminant diets [16]. Gross energy was neither measured nor used in the calculation of DE or ME or in the statistical analyses.

### 2.8. Growth Performance

Body weight (BW) was recorded on Days 0, 30, 60, and 90 of the experimental period. Before each weighing, feed was withheld for 16 h, whereas drinking water remained available. Goats were weighed individually in the morning before feeding and at approximately the same time of day to minimize variation associated with feeding and gastrointestinal fill.

Body weight was measured using a digital hanging scale (WH-C100; WeiHeng, Guangzhou, China) with a maximum capacity of 150 kg and a readability of 0.05 kg within the body-weight range of the experimental goats. Each goat was placed securely in a livestock weighing sling attached to the scale. The sling was suspended freely without contacting the floor or surrounding structures, and its weight was tared before each measurement. Before each weighing session, the scale was zeroed, and its readings were checked using standard weights. Body weight was recorded after the displayed value had stabilized.

Total body-weight gain was calculated as the difference between BW on Days 90 and 0. Average daily gain (ADG) was calculated by dividing total body-weight gain by the 90-day experimental period. Mean daily DMI was calculated for each goat over the experimental period as described in Section 2.7 and included dry matter consumed from both the basal TMR and the supplemented hydrolysate. The gain-to-feed ratio (G:F) was calculated as ADG divided by mean daily DMI and expressed as kilograms of body-weight gain per kilogram of dry matter consumed.

### 2.9. Blood Collection and Processing

Blood was collected by jugular venipuncture before the morning feeding on Day 0, before the initiation of supplementation, to assess baseline health status. These measurements were collected as clinical screening data and were not prespecified as part of the longitudinal evaluation of treatment responses. Accordingly, Day 0 values were summarized descriptively but were not included in the inferential analyses of Day 90 outcomes.

On Day 90, blood was collected immediately before the morning feeding (0 h; preprandial) and 4 h after feeding. The preprandial sample was obtained to minimize the immediate influence of the morning meal, whereas the 4-h sample represented a standardized postprandial period during active digestion and nutrient absorption. Sampling at both time points allowed the effects of treatment, sampling time, and their interaction on hematological and serum biochemical outcomes to be evaluated while accounting for short-term variation associated with feeding. The 4-h time point was not intended to represent a confirmed peak response. Immunoglobulin, cytokine, and gene-expression analyses were performed using only the Day 90 preprandial sample.

Before venipuncture, the collection site was disinfected with 70% ethanol. Blood was collected by trained personnel using a sterile 20-gauge, 1.5-inch needle. The blood-collection procedures and reporting are described in accordance with the ARRIVE 2.0 guidelines [15].

For hematological analysis, approximately 3 mL of blood were collected into a dipotassium ethylenediaminetetraacetic acid (K_2_EDTA) tube at each applicable sampling time. Samples were maintained at 4 °C and analyzed within 2 h of collection. For peripheral blood leukocyte (PBL) isolation, an additional 6 mL of blood were collected into a separate K_2_EDTA tube from the Day 90 preprandial sample. This sample was maintained at room temperature and processed within 2 h of collection. PBLs were isolated as described in Section 2.12, washed, transferred into RNA lysis buffer, and stored at −80 °C until RNA extraction.

For serum preparation, approximately 5 mL of blood were collected into a plain tube at each applicable sampling time and allowed to clot at room temperature for 30 min. Samples were subsequently centrifuged at 1500× *g* for 15 min at 4 °C. Serum was divided into 0.5-mL aliquots and stored at −80 °C until serum biochemical, immunoglobulin, and cytokine analyses. Separate aliquots were used for the respective assays, and no aliquot underwent more than one freeze–thaw cycle.

### 2.10. Hematological and Serum Biochemical Analyses

Whole blood collected in K_2_EDTA tubes was gently mixed and analyzed within 2 h of collection using an automated veterinary hematology analyzer (BC-30 Vet; Shenzhen Mindray Animal Medical Technology Co., Ltd., Shenzhen, China) operated with the goat-specific setting. The reported hematological variables included red blood cell count (RBC), white blood cell count (WBC), mean corpuscular volume (MCV), mean corpuscular hemoglobin (MCH), mean corpuscular hemoglobin concentration (MCHC), and the percentages of neutrophils, lymphocytes, and monocytes. RBC was reported in ×10^6^/µL, WBC in ×10^3^/µL, MCV in fL, MCH in pg, MCHC in g/dL, and differential leukocytes as percentages of total WBC. The erythrocyte indices and leukocyte differential percentages were calculated automatically by the analyzer according to the manufacturer’s algorithms.

Serum samples were thawed once at 4 °C and analyzed for total protein, aspartate aminotransferase (AST), alanine aminotransferase (ALT), and alkaline phosphatase (ALP) using commercial colorimetric reagent kits (BioSystems S.A., Barcelona, Spain) according to the manufacturers’ instructions. Absorbance was measured using a C-7100 UV–visible spectrophotometer (PEAK Instruments Inc., Houston, TX, USA). Total protein was determined using the Biuret endpoint method, AST and ALT activities using kinetic ultraviolet methods, and ALP activity using a kinetic p-nitrophenyl phosphate method. Total protein was reported in g/dL, whereas enzyme activities were reported in U/L. Samples were analyzed in duplicate and, whenever possible, within the same analytical run to minimize inter-assay variation.

### 2.11. Serum Immunoglobulin and Cytokine Analyses

Serum immunoglobulin and cytokine concentrations were determined using enzyme-linked immunosorbent assays (ELISAs) according to the respective manufacturers’ instructions and established ELISA principles [19]. Only preprandial serum samples collected on Day 90 were used for these analyses.

#### 2.11.1. Serum Immunoglobulins

Serum IgA, IgG, and IgM concentrations were determined using quantitative sandwich ELISA kits specified by their manufacturers as reactive with goat serum. IgA was measured using a Goat IgA ELISA Kit (catalogue no. A80214; Antibodies.com, Cambridge, UK), with a calibration range of 0.313–20 ng/mL and a manufacturer-reported sensitivity of 0.188 ng/mL. IgG was measured using a Goat IgG ELISA Kit (Colorimetric; catalogue no. NBP3-12520; Novus Biologicals, a Bio-Techne brand, Minneapolis, MN, USA), with a calibration range of 0.69–500 ng/mL. IgM was measured using a Goat IgM ELISA Kit (catalogue no. A80215; Antibodies.com), with a calibration range of 0.234–15 ng/mL and a manufacturer-reported sensitivity of 0.141 ng/mL.

Serum samples were initially diluted 1:100,000 for IgA, 1:500,000 for IgG, and 1:200,000 for IgM using the sample diluent supplied or specified for the respective kit. Additional dilution was performed when necessary to bring sample responses within the corresponding calibration range. Final concentrations were corrected for the applicable dilution factors. For the IgA and IgM kits, the manufacturer-reported intra- and inter-assay coefficients of variation were <8% and <10%, respectively.

#### 2.11.2. Serum Cytokines

Serum IL-1β, IL-6, and IL-10 concentrations were determined using ELISA kits specified by their manufacturers as reactive with goat serum. IL-1β was measured using a quantitative sandwich ELISA kit (catalogue no. CSB-E16568G; CUSABIO Technology LLC, Houston, TX, USA), with a calibration range of 3.12–200 pg/mL and a manufacturer-reported sensitivity of 0.78 pg/mL. The manufacturer-reported intra- and inter-assay coefficients of variation were <8% and <10%, respectively.

IL-6 was measured using a quantitative competitive ELISA kit (catalogue no. MBS734666; MyBioSource, Inc., San Diego, CA, USA), with a stated calibration range of 7.8–500 pg/mL and a manufacturer-reported sensitivity of 1.0 pg/mL. IL-10 was measured using a quantitative sandwich ELISA kit (catalogue no. MBS7606891; MyBioSource, Inc.), with a calibration range of 15.625–1000 pg/mL and a manufacturer-reported sensitivity of 9.375 pg/mL. The manufacturer-reported intra- and inter-assay coefficients of variation for these kits were <8% and <10%, respectively.

Cytokine assays were initially performed using undiluted serum. Samples with responses above the upper limit of the applicable calibration range, including selected IL-1β samples, were diluted 1:2 using the respective sample diluent, reassayed, and corrected for the dilution factor.

Standards, blanks, assay controls, and serum samples were analyzed in duplicate. A separate standard curve was generated for each assay run, and sample concentrations were calculated only when their responses fell within the range bracketed by the corresponding standards. Absorbance was measured at 450 nm using a Multiskan FC microplate photometer (Thermo Fisher Scientific, Waltham, MA, USA). An individual sample was reassayed when the coefficient of variation between duplicate wells exceeded 10% or when its response fell outside the calibration range. An assay run was repeated when the standard curve or assay controls failed to meet the manufacturer-specified acceptance criteria.

The calibration ranges, analytical sensitivities, and intra- and inter-assay precision values reported above were obtained from the respective manufacturers’ documentation and were not independently reproduced in this study. Although standard-curve performance and duplicate agreement were monitored as within-run quality-control measures, no independent matrix-specific validation, including dilutional parallelism, spike-and-recovery testing, or formal assessment of matrix effects, was conducted using serum from the experimental goats. Manufacturer-reported performance characteristics should therefore not be interpreted as in-house validation of these assays for the present study matrix.

### 2.12. RNA Extraction and One-Step RT-qPCR

Peripheral blood leukocytes (PBLs) isolated from K_2_EDTA-anticoagulated blood were used for gene-expression analysis. Erythrocytes were removed using a laboratory-prepared ammonium–chloride–potassium red blood cell lysis buffer containing 155 mM ammonium chloride, 10 mM potassium bicarbonate, and 0.1 mM EDTA, adjusted to pH 7.2–7.4. Briefly, blood was mixed with three volumes of lysis buffer, incubated at room temperature for 10 min with occasional gentle inversion, and centrifuged at 400× *g* for 10 min at 4 °C. The supernatant was discarded, and the leukocyte pellet was washed twice with sterile phosphate-buffered saline. The erythrocyte-lysis step was repeated once when visible erythrocyte contamination remained. The resulting leukocyte pellet was either processed immediately or suspended in the RNA-extraction kit lysis buffer and stored at −80 °C until RNA extraction.

Total RNA was extracted using the RNeasy Plus Mini Kit (catalogue no. 74134; QIAGEN, Hilden, Germany) according to the manufacturer’s instructions. Genomic DNA was removed using the gDNA Eliminator Mini Spin Columns supplied with the kit; therefore, no separate DNase treatment was performed. RNA concentration and spectrophotometric purity were assessed using a MaestroNano MN-917 microvolume spectrophotometer (Maestrogen Inc., Hsinchu, Taiwan). Samples with A_260_/A_280_ ratios of 1.8–2.1 were accepted for analysis. RNA samples were normalized to 1 ng/µL using nuclease-free water and stored at −80 °C until one-step reverse-transcription quantitative PCR (RT-qPCR).

RNA integrity was not assessed using gel electrophoresis or an automated electrophoretic platform; consequently, RNA integrity numbers were not available. The A_260_/A_280_ ratio is therefore reported only as an indicator of spectrophotometric purity and should not be interpreted as evidence of RNA integrity.

Gene-expression experiments were conducted and reported with reference to the Minimum Information for Publication of Quantitative Real-Time PCR Experiments (MIQE) guidelines [20]. One-step RT-qPCR was performed using the SensiFAST SYBR No-ROX One-Step Kit (catalogue no. BIO-72005; Bioline, London, UK) on a CFX96 Real-Time PCR Detection System (Bio-Rad Laboratories, Hercules, CA, USA). Each 20-µL reaction contained 10 µL of 2× SensiFAST SYBR No-ROX One-Step Mix, forward and reverse primers at final concentrations of 200 nM each, 0.2 µL of reverse transcriptase, 0.4 µL of RiboSafe RNase inhibitor, 4 µL of normalized RNA template corresponding to 4 ng of total RNA, and nuclease-free water to the final reaction volume.

Thermal cycling consisted of reverse transcription at 45 °C for 10 min, polymerase activation at 95 °C for 2 min, and 40 amplification cycles at 95 °C for 5 s and 60 °C for 20 s. A melting-curve analysis was subsequently performed from 65 to 95 °C in increments of 0.5 °C. Each biological sample was analyzed in technical duplicate. A no-template control, in which the RNA template was replaced with nuclease-free water, and a no-reverse-transcription control, prepared without reverse transcriptase, were included for each primer pair. The presence of a single melting peak and the absence of amplification in the negative controls were used as indicators of amplification specificity and absence of detectable contamination. These quality-control procedures were not considered substitutes for direct assessment of RNA integrity or sequence-based confirmation of the amplicons.

The investigated genes comprised innate-recognition and signaling genes (*TLR4*, *NFKB1*, and *NFKBIA*), pro-inflammatory genes (*TNF*, *IL1B*, and *IL6*), and regulatory genes (*IL10* and *TGFB1*). *GAPDH* was used as the reference gene. Amplification efficiencies were evaluated using five-point, 10-fold serial dilutions of pooled RNA, with each dilution subjected to the complete one-step RT-qPCR procedure. Primer pairs were accepted when amplification efficiencies were 90–110%, standard-curve coefficients of determination were R^2^ ≥ 0.98, a single melting peak was observed, and no amplification occurred in the negative controls.

Relative gene expression was calculated using the 2^−ΔΔCq^ method [21]. For each sample, the Cq value of the target gene was normalized to the corresponding *GAPDH* Cq value to obtain ΔCq. The mean ΔCq of the CON group on Day 90 was used as the calibrator, and results were expressed as fold changes relative to this group. Primer sequences, accession numbers, amplicon sizes, and amplification efficiencies are provided in Supplementary Table S1.

### 2.13. Rumen Fluid Collection

Rumen fluid was collected once from each goat on Day 90, approximately 3 h after the morning feeding. This sampling time was selected to standardize the interval between feeding and sample collection and to obtain an endpoint measurement during the active postprandial fermentation period. Samples were collected using a flexible oral stomach tube connected to a portable vacuum pump, following a previously described stomach-tubing approach for sheep and goats [22].

The tube was approximately 10–12 mm in external diameter, contained multiple openings near its distal end, and was inserted approximately 120–150 cm from the incisors to position the sampling end within the rumen. Placement was assessed based on the absence of respiratory sounds or coughing during aspiration and the characteristic appearance and odor of the aspirated rumen fluid.

First, approximately 50 mL of aspirated fluid were discarded to reduce potential contamination with saliva and esophageal contents. Approximately 100 mL of the subsequent rumen fluid were collected from each goat into a clean container. When obvious salivary contamination was suspected based on a clear, viscous appearance and an atypically high initial pH, the sample was discarded, and collection was repeated. The stomach tube and collection apparatus were thoroughly rinsed and disinfected between animals to minimize carryover and cross-contamination.

Immediately after collection, rumen fluid was filtered through four layers of cheesecloth. The pH of the filtered fluid was measured within 10 min of collection using a FiveEasy F20 benchtop pH meter equipped with an LE407 electrode (Mettler-Toledo GmbH, Greifensee, Switzerland). The instrument was calibrated immediately before the sampling session using standard buffer solutions at pH 4.00 and 7.00 at room temperature. The electrode was rinsed with distilled water and gently blotted dry between samples. Two consecutive readings were obtained, and the measurement was accepted when the readings differed by no more than 0.05 pH units. After pH measurement, rumen-fluid subsamples were processed for volatile fatty acid analysis as described in Section 2.14.

Because rumen fluid was collected only once from each goat, without a pretreatment baseline or repeated sampling across the day, the measurements represent a single postprandial endpoint. They cannot characterize within-animal changes from baseline, diurnal variation, or the persistence of the observed differences. In addition, oral stomach-tube sampling may be influenced by residual salivary contamination and variation in the ruminal location from which fluid is aspirated.

### 2.14. Analysis of Acetate, Propionate, and Butyrate

Immediately after pH measurement, four volumes of filtered rumen fluid were mixed with one volume of 25% (*w*/*v*) metaphosphoric acid to precipitate proteins and inhibit further microbial activity, following the general sample-preparation approach described by Erwin et al. [23]. The metaphosphoric acid solution contained 10 mM 2-ethylbutyric acid as an internal standard, resulting in a final internal-standard concentration of 2 mM in the acidified samples. Samples were mixed thoroughly, maintained at 4 °C for at least 30 min, and centrifuged at 10,000× *g* for 15 min at 4 °C. The resulting supernatant was filtered through a 0.22-µm polytetrafluoroethylene syringe filter into an autosampler vial and stored at −20 °C until analysis. Samples were thawed only once before injection.

Acetate, propionate, and butyrate were the only volatile fatty acids (VFAs) quantified in this study. Their combined concentration was calculated as the sum of these three measured VFAs and is hereafter referred to as the “sum of measured VFAs.” This value does not represent the total concentration of all VFAs present in rumen fluid.

Acetate, propionate, and butyrate concentrations were determined by gas chromatography with flame-ionization detection (GC–FID) using a 7890B GC system (Agilent Technologies, Santa Clara, CA, USA). Separation was performed using a DB-FFAP free-fatty-acid-phase capillary column (30 m × 0.25 mm internal diameter, 0.25 µm film thickness; Agilent Technologies). Helium was used as the carrier gas at a constant flow rate of 1.2 mL/min. Injector and detector temperatures were maintained at 250 and 280 °C, respectively. The initial oven temperature was maintained at 100 °C for 1 min, increased to 180 °C at 10 °C/min, and then maintained at 180 °C for 2 min. A 1-µL aliquot of each prepared sample was injected using a split ratio of 20:1.

Analytical standards of acetic acid (catalogue no. 71251; CAS no. 64-19-7), propionic acid (catalogue no. 94425; CAS no. 79-09-4), and butyric acid (catalogue no. 19215; CAS no. 107-92-6) were obtained from Sigma-Aldrich (St. Louis, MO, USA). Individual stock solutions were used to prepare a mixed-standard series. After undergoing the same metaphosphoric-acid treatment as the rumen-fluid samples, the prepared calibration standards contained acetate, propionate, and butyrate at final concentrations of 5–125, 2–50, and 1–25 mM, respectively. The calibration levels maintained approximate acetate-to-propionate-to-butyrate proportions of 5:2:1. A reagent blank was processed in parallel. Calibration standards and samples contained 2-ethylbutyric acid at the same final concentration of 2 mM.

Calibration curves included at least six non-zero concentrations and were constructed by plotting the analyte-to-internal-standard peak-area ratio against the corresponding analyte concentration. A calibration curve was accepted when its coefficient of determination was R^2^ ≥ 0.995, and the back-calculated concentrations of the standards were within ±15% of their nominal values. Samples with analyte responses above the calibration range were reanalyzed after appropriate dilution using an acidified diluent containing 2-ethylbutyric acid at the same final concentration as the prepared samples.

Peaks were identified by comparing their retention times with those of the corresponding authentic standards. Concentrations were calculated from the internal-standard calibration curves and corrected for dilution introduced during sample preparation. Individual VFA concentrations were reported as mmol/L of the original rumen-fluid sample. The sum of measured VFAs was calculated as the sum of acetate, propionate, and butyrate concentrations. The acetate-to-propionate ratio was calculated by dividing the acetate concentration by the propionate concentration.

### 2.15. Statistical Analysis

Statistical analyses, except for gene-expression outcomes, were performed using R version 4.5.1 (R Foundation for Statistical Computing, Vienna, Austria). The individual goat was the experimental unit. Linear mixed-effects models were fitted by restricted maximum likelihood using the nlme package (version 3.1.168) [24]. Treatment was included as a fixed effect and initial-body-weight block was included as a random intercept because it represented a design factor used to account for nuisance variation associated with initial body weight. With only five blocks, however, the estimated block-level variance may have been imprecise.

Single-time-point outcomes were analyzed using models containing treatment as a fixed effect and block as a random effect. Body weight measured on Days 0, 30, 60, and 90 was analyzed using a repeated-measures model containing treatment, experimental day, and treatment × experimental-day interaction as fixed effects. Day 90 hematological and serum biochemical outcomes were analyzed using repeated-measures models containing treatment, sampling time (0 and 4 h), and treatment × sampling-time interaction as fixed effects. For both repeated-measures analyses, block was included as a random effect, goat was the repeated subject, and a compound-symmetry correlation structure was used. When an interaction was detected, treatment effects were examined separately within each sampling time using Tukey-adjusted comparisons; otherwise, the overall treatment effect across sampling times was interpreted.

Day 0 hematological and serum biochemical measurements were collected for health screening and are presented descriptively by treatment in Supplementary Table S3. They were not included as repeated observations or covariates in the Day 90 models because they were not prespecified as longitudinal analytical baseline outcomes. No hypothesis tests of baseline balance were performed.

Model assumptions were assessed using residual-versus-fitted and Q–Q plots and the Shapiro–Wilk test of residuals. Outcomes were log_10_- or square-root-transformed when necessary. Estimated marginal means, model-based 95% confidence intervals, and Tukey-adjusted comparisons were obtained using the emmeans package (version 2.0.4).

Linear and quadratic dose-response contrasts were evaluated using the actual supplementation levels of 0, 2.5, 5.0, and 10.0 g/goat/day, with coefficients of −7, −3, 1, and 9 and 7, −4, −8, and 5, respectively. Because supplementation was administered as a fixed amount per goat, these contrasts represent responses across daily amounts rather than constant body-weight-normalized doses.

Gene-expression outcomes were analyzed using GraphPad Prism version 11.0.2 (GraphPad Software, Boston, MA, USA) using a randomized-block one-way analysis matched by initial-body-weight block. Tukey-adjusted pairwise comparisons and linear and quadratic dose-response contrasts were evaluated. Gene-expression figures present individual biological observations with the mean ± SD.

The Benjamini–Hochberg procedure was applied within prespecified outcome families comprising growth and intake, hematology, serum biochemistry, circulating immune indicators, immune-related gene expression, and ruminal measures [25]. Within each family, FDR adjustment was performed separately for the relevant overall treatment effects, linear contrasts, quadratic contrasts, sampling-time effects, and treatment × sampling-time interactions. Both unadjusted *p*-values and FDR-adjusted *q*-values are reported; *q* < 0.05 indicated statistical significance after multiplicity correction. Exact *p*-values and model-based 95% confidence intervals are reported where applicable. Nonsignificant results were not interpreted as evidence of equivalence or absence of an effect.

## 3. Results

### 3.1. Yield and Characteristics of the MOS-Rich Hydrolysate

The production process yielded 12.58 ± 0.42 g of freeze-dried hydrolysate per 100 g of initial PKM dry matter (Table 2). The hydrolysate contained 4.82 ± 0.21% moisture on an as-is basis. On a dry matter basis, the total carbohydrate and reducing sugar contents, expressed as D-mannose equivalents, were 72.46 ± 1.83% and 58.27 ± 1.46%, respectively. Crude protein and ash accounted for 6.35 ± 0.32% and 5.18 ± 0.27% of dry matter, respectively. The unaccounted fraction, calculated by difference from total carbohydrate, crude protein, and ash, was 16.01 ± 1.88% of dry matter. Reducing sugars were included within the total-carbohydrate measurement and therefore represented an overlapping analytical measure rather than an additional compositional fraction.

The hydrolysate exhibited a water solubility of 93.64 ± 1.12% of the initial dry sample weight, and its 1% (*w*/*v*) aqueous solution had a pH of 5.62 ± 0.08. These results indicate that the recovered hydrolysate had high total-carbohydrate content and was predominantly water-soluble; however, the colorimetric carbohydrate assays did not distinguish individual sugars or oligosaccharides.

### 3.2. HPLC–RID Identification and Quantitative MOS Composition

HPLC–RID analysis detected peaks with retention times corresponding to mannobiose (M2), mannotriose (M3), mannotetraose (M4), mannopentaose (M5), and mannohexaose (M6) (Figure S1 and Table 3). Sample retention times differed from those of the corresponding authentic standards by only 0.24–0.33%, remaining within the predefined acceptance criterion of ±2%. The compounds were therefore assigned as retention-time-matched MOS fractions. Because identification was based on retention-time comparison using refractive index detection, the assignments should be considered tentative rather than structurally confirmed.

The total identified concentration of M2–M6 was 534.6 ± 8.6 mg/g hydrolysate, corresponding to 53.46 ± 0.86% (*w*/*w*) of the freeze-dried product. M2 was the predominant fraction, with a concentration of 205.0 ± 4.1 mg/g hydrolysate, and accounted for 38.35% of total MOS. M3 was the second most abundant fraction at 145.0 ± 3.8 mg/g and represented 27.12% of total MOS. Together, M2 and M3 accounted for 65.47% of the identified MOS.

The concentrations of the higher-degree-of-polymerization fractions decreased progressively from M4 to M6. M4, M5, and M6 were present at 95.0 ± 3.5, 55.0 ± 4.0, and 34.6 ± 3.7 mg/g hydrolysate, respectively, corresponding to 17.77%, 10.29%, and 6.47% of total MOS. Overall, the hydrolysate was characterized by a predominance of short-chain MOS, particularly M2 and M3, with progressively lower contributions from M4–M6.

### 3.3. Feed Intake and Growth Performance

All 20 goats completed the 90-day experiment. No animal met the prespecified withdrawal criteria or required veterinary intervention considered related to supplementation. The unadjusted mean initial BW was 17.46, 17.54, 17.48, and 17.52 kg in the CON, MOS2.5, MOS5, and MOS10 groups, respectively (Table 4). Initial BW is presented descriptively as mean ± SD because it was used to construct the blocks before treatment allocation and was not considered a treatment-response outcome.

No statistically significant linear or quadratic dose-response associations were detected for final BW, total BW gain, ADG, DMI, G:F, or the intakes of OM, CP, NDF, and ADF (Table 4). NDF intake showed a nominal positive linear trend with supplementation level (raw *p* = 0.056), without evidence of a quadratic association (raw *p* = 0.922); however, this association did not meet the conventional significance threshold and was not significant after FDR correction. None of the feed-intake or growth outcomes remained statistically significant after correction for multiple testing. Although final BW and ADG were numerically higher in the MOS5 and MOS10 groups than in the CON group, these differences were not statistically significant and should not be interpreted as evidence of improved growth performance.

### 3.4. Hematological and Serum Biochemical Indices

Available Day 0 hematological and serum biochemical values are presented descriptively in Table S3. The distributions broadly overlapped among treatment groups, although numerical differences were observed for some variables, including MCV, MCH, WBC, and AST. Because these measurements were collected for health screening rather than as a prespecified longitudinal baseline, no formal baseline comparisons were performed, and the values were not included in the inferential analyses of the Day 90 outcomes. Given the small number of goats per treatment, the descriptive overlap should not be interpreted as evidence of baseline equivalence.

#### 3.4.1. Hematology

Hematological indices measured before feeding and 4 h after feeding on Day 90 are presented in Table 5. For outcomes without evidence of a treatment × sampling-time interaction, MCV showed a negative linear association with supplementation level (raw *p* < 0.001), whereas MCHC showed a nominal quadratic association (raw *p* = 0.030). No statistically significant dose-response association was detected for RBC or WBC. Sampling time was not associated with RBC, MCV, or WBC (raw *p* ≥ 0.080), whereas MCHC was higher 4 h after feeding than before feeding (raw *p* = 0.020). The corresponding FDR-adjusted results are provided in Table 5.

A treatment × sampling-time interaction was detected nominally for MCH (raw *p* = 0.030), but this interaction did not remain statistically significant after FDR correction (*q* = 0.080). Accordingly, the time-specific MCH results were interpreted cautiously. Before feeding, the linear contrast was not statistically significant (raw *p* = 0.080; *q* = 0.128), whereas the quadratic contrast remained significant after FDR correction (raw *p* < 0.001; *q* ≤ 0.003). At 4 h after feeding, the linear contrast remained significant (raw *p* = 0.010; *q* = 0.027), whereas the quadratic contrast did not (raw *p* = 0.550; *q* = 0.640).

Neutrophil and lymphocyte percentages differed between the two sampling times. Across treatments, neutrophil percentages were higher, and lymphocyte percentages were lower 4 h after feeding than before feeding. The sampling-time effects remained statistically significant after FDR correction for both neutrophils and lymphocytes (raw *p* < 0.001; *q* ≤ 0.003 for each). Treatment × sampling-time interactions also remained significant for neutrophils (raw *p* = 0.003; *q* = 0.012) and lymphocytes (raw *p* < 0.001; *q* ≤ 0.008), indicating that the magnitude or pattern of the differences between the two sampling times varied among treatments.

Before feeding, neutrophil percentage showed significant linear and quadratic dose-response contrasts after FDR correction (linear: raw *p* = 0.020, *q* = 0.040; quadratic: raw *p* < 0.001, *q* ≤ 0.003). At 4 h after feeding, the linear contrast remained significant (raw *p* = 0.010; *q* = 0.027), whereas the quadratic contrast did not (raw *p* = 0.080; *q* = 0.320). Lymphocyte percentage showed a significant linear contrast before feeding (raw *p* = 0.010; *q* = 0.027). At 4 h after feeding, neither the linear nor the quadratic contrast remained significant after FDR correction (linear: raw *p* = 0.330, *q* = 0.440; quadratic: raw *p* = 0.010, *q* = 0.080).

Collectively, these findings demonstrate sampling-time-dependent differences in relative leukocyte distributions, with treatment-related patterns that were not uniform across the two sampling times. The results describe between-group differences at the Day 90 endpoint and should not be interpreted as within-animal changes from baseline.

#### 3.4.2. Serum Biochemistry

Serum biochemical indices measured before feeding and 4 h after feeding on Day 90 are presented in Table 6. No statistically significant treatment × sampling-time interaction was detected for total protein, AST, ALT, or ALP (raw *p* ≥ 0.360). Accordingly, treatment effects for these outcomes were interpreted across the two sampling times. No statistically significant treatment effect or linear or quadratic dose-response association was detected for any of the evaluated serum biochemical indices after FDR correction.

Sampling time was not associated with total protein or ALT (raw *p* = 0.860 and *p* = 0.720, respectively). AST and ALP activities were numerically higher 4 h after feeding than before feeding, with nominal sampling-time effects for AST (raw *p* = 0.040) and ALP (raw *p* = 0.020). These sampling-time effects did not remain statistically significant after correction for multiple testing (FDR-adjusted *q* = 0.080 for both AST and ALP). 

In the time-specific analysis, ALP activity at 4 h showed a nominal negative linear association with supplementation level (raw *p* = 0.070), whereas the linear contrast for the mean across sampling times was not statistically significant (raw *p* = 0.120). Neither association remained statistically significant after FDR correction. Complete estimated marginal means at 0 and 4 h after feeding are provided in Supplementary Table S5.

### 3.5. Circulating Immunoglobulins and Cytokines

Serum immunoglobulin and cytokine concentrations measured at the Day 90 endpoint are presented in Table 7. IgA, IgG, IgM, IL-1β, IL-6, and IL-10 were treated as a prespecified family of six circulating immune indicators for multiplicity adjustment.

After Benjamini–Hochberg FDR correction, serum IgA showed a significant positive linear association with the assigned supplementation level (raw *p* = 0.018; *q* = 0.036). Serum IL-1β and IL-6 showed significant negative linear associations (IL-1β: raw *p* = 0.003, *q* = 0.012; IL-6: raw *p* = 0.004, *q* = 0.012). The nominal positive linear association for IL-10 did not reach statistical significance either before or after FDR correction (raw *p* = 0.061; *q* = 0.091). No significant linear association was detected for IgG or IgM, and none of the quadratic contrasts remained significant after FDR correction.

Because pretreatment immunoglobulin and cytokine concentrations were not measured, these findings represent between-group differences at the Day 90 endpoint and do not demonstrate within-animal changes from baseline.

### 3.6. Immune-Related Gene Expression

Immune-related gene expression in peripheral blood leukocytes was evaluated using preprandial samples collected at the Day 90 endpoint. The eight gene-expression outcomes were treated as a distinct transcriptional outcome family, and Benjamini–Hochberg FDR correction was applied separately to the linear and quadratic contrasts across these genes. Complete raw *p*-values, FDR-adjusted *q*-values, and Tukey-adjusted pairwise comparisons are provided in Supplementary Table S2.

Among genes associated with innate immune recognition and NF-κB-related signaling, the relative expression of *TLR4*, *NFKB1*, and *NFKBIA* showed significant negative linear associations with the assigned supplementation level (Figure 1). The corresponding raw *p*-values and FDR-adjusted *q*-values were 0.002 and 0.004 for *TLR4*, 0.003 and 0.005 for *NFKB1*, and 0.004 and 0.005 for *NFKBIA*, respectively. None of the quadratic contrasts remained significant after FDR correction (*q* ≥ 0.315). Tukey-adjusted pairwise comparisons showed lower relative expression in MOS5 and MOS10 than in CON, whereas MOS2.5 generally had intermediate values.

The relative expression of the pro-inflammatory genes *TNF*, *IL1B*, and *IL6* also showed significant negative linear associations with supplementation level (Figure 2A–C). The raw *p*-values and FDR-adjusted *q*-values were 0.001 and 0.004 for *TNF*, 0.001 and 0.004 for *IL1B*, and 0.002 and 0.004 for *IL6*, respectively. None of the corresponding quadratic contrasts remained significant after FDR correction (*q* ≥ 0.315). Relative expression was numerically lowest in MOS10, with the lower-dose groups generally showing intermediate values; gene-specific Tukey-adjusted pairwise comparisons are reported in Supplementary Table S2.

In contrast, *IL10* and *TGFB1* showed significant positive linear associations with supplementation level (Figure 3A,B). For *IL10*, the raw linear *p*-value was 0.008, and the FDR-adjusted *q*-value was 0.008. Tukey-adjusted comparisons showed higher relative expression in MOS5 and MOS10 than in CON. For *TGFB1*, the raw linear *p*-value was 0.005, and the FDR-adjusted *q*-value was 0.006, with the highest numerical value observed in MOS10. The nominal quadratic association for *IL10* did not remain significant after FDR correction (raw *p* = 0.064; *q* = 0.315).

These findings represent between-group differences in peripheral blood leukocyte gene expression at the Day 90 endpoint. Because pretreatment gene expression was not measured, they do not demonstrate within-animal changes over time or establish coordinated regulation of an immune-signaling pathway.

### 3.7. Ruminal pH and Measured VFA Profile

Ruminal pH and the concentrations of acetate, propionate, and butyrate were determined from a single rumen-fluid sample collected 3 h after feeding on Day 90 (Table 8). Ruminal pH, acetate concentration, and the sum of the measured VFA concentrations showed no statistically significant linear or quadratic associations with the assigned supplementation level, either before or after FDR correction.

Before multiplicity adjustment, propionate and butyrate concentrations showed nominal positive linear associations with supplementation level (raw *p* = 0.028 and 0.021, respectively), whereas the acetate-to-propionate ratio showed a nominal negative linear association (raw *p* = 0.018). However, none of these linear associations remained statistically significant after FDR correction within the ruminal-outcome family (*q* = 0.056 for each outcome).

Within-outcome Tukey-adjusted pairwise comparisons showed a higher propionate concentration in MOS2.5 than in CON, whereas MOS5 and MOS10 had intermediate values. Butyrate concentration was higher in MOS10 than in CON, with intermediate values in MOS2.5 and MOS5. The acetate-to-propionate ratio was lower in MOS2.5 than in CON, whereas MOS5 and MOS10 had intermediate values. These pairwise differences should be interpreted as exploratory because the corresponding dose-response associations did not remain significant after correction across the ruminal outcomes.

No quadratic association was detected for propionate (raw *p* = 0.164) or the acetate-to-propionate ratio (raw *p* = 0.237). Butyrate showed a nominal quadratic association before multiplicity adjustment (raw *p* = 0.081), but this association was not statistically significant before or after FDR correction. Because rumen fluid was collected at only one postprandial time point, these results represent between-group endpoint differences in the measured VFA profile and do not demonstrate within-animal changes or a sustained alteration in ruminal fermentation.

## 4. Discussion

### 4.1. Principal Findings

The present study identified dose-related between-group differences in selected circulating and transcriptional immune indicators at the Day 90 endpoint. Within the circulating immune-indicator family, serum IgA showed a positive linear association with the assigned supplementation level, whereas IL-1β and IL-6 showed negative linear associations. These associations remained statistically significant after FDR correction. However, because pretreatment immunoglobulin and cytokine concentrations were not measured, the findings do not demonstrate within-animal increases or decreases during the supplementation period. Pre-existing group differences also cannot be excluded, particularly given the limited number of goats per treatment, despite randomized allocation within initial-body-weight blocks.

The peripheral blood leukocyte gene-expression results showed negative linear associations for *TLR4*, *NFKB1*, *NFKBIA*, *TNF*, *IL1B*, and *IL6* and positive linear associations for *IL10* and *TGFB1*. These associations remained statistically significant after FDR correction across the eight-gene outcome family. Nevertheless, the results represent endpoint differences rather than treatment-induced transcriptional changes because pretreatment gene expression was not measured. Moreover, circulating proteins and leukocyte transcripts represent distinct biological levels, and their relationships were not evaluated using correlation, mediation, or pathway analyses.

The broadly corresponding directions of selected circulating and transcriptional indicators are compatible with between-group differences in inflammation-related measures, but they do not establish attenuation of TLR4/NF-κB signaling or coordinated immunological regulation. Receptor activation, NF-κB nuclear translocation, protein phosphorylation, downstream protein abundance, and immune-cell function were not measured. In addition, because gene expression was evaluated in mixed peripheral blood leukocytes, the observed differences may reflect variation in leukocyte composition, transcript abundance within specific leukocyte populations, or both.

At the single Day 90 rumen-fluid sampling, propionate and butyrate concentrations showed nominal positive linear associations with supplementation level, whereas the acetate-to-propionate ratio showed a nominal negative linear association. However, these associations did not remain statistically significant after FDR correction within the ruminal-outcome family. Ruminal pH, acetate concentration, and the sum of the measured VFA concentrations showed no detectable dose-response associations. These findings therefore provide preliminary endpoint evidence concerning the measured VFA profile and do not establish a sustained alteration in ruminal fermentation.

Feed intake and growth performance did not differ detectably among treatments. Overall, the findings provide preliminary evidence that graded supplementation with the complete PKM-derived hydrolysate was associated with selected immune-related endpoint measures. Because the hydrolysate was chemically heterogeneous, the observed associations cannot be attributed specifically to the identified M2–M6 oligosaccharides. Confirmation requires larger, adequately powered studies incorporating pretreatment immune measurements, repeated sampling, independently validated assays, prospectively designated primary outcomes, and more comprehensive characterization of ruminal fermentation and microbial responses.

### 4.2. Circulating and Transcriptional Immune Indicators

The linear endpoint associations for IgA, IL-1β, and IL-6 remained statistically significant after FDR correction across the six circulating immune indicators, reducing, but not eliminating, the possibility that these findings resulted from multiple testing. Nevertheless, the limited sample size, absence of pretreatment immune measurements, and lack of independent matrix-specific validation of the ELISA kits constrain causal and mechanistic interpretation. The findings therefore concern between-group differences at the Day 90 endpoint rather than confirmed within-animal changes during supplementation.

Serum IgA showed a positive linear association with the assigned supplementation level. IgA has important roles in mucosal immune surveillance, including immune exclusion, antigen neutralization, and regulation of host–microbiota interactions [26]. Previous studies have reported differences in immunoglobulin concentrations, intestinal immune indicators, or related physiological measures following supplementation with MOS, mannan-rich fractions, or multicomponent prebiotics; however, responses have varied with animal age, product composition, dose, physiological status, and experimental context [1,27,28]. In the present study, no significant dose-response association was detected for IgG or IgM. Although the observed pattern was therefore confined to IgA among the measured immunoglobulins, the absence of detectable associations for IgG and IgM does not establish a selective biological effect, particularly given the limited statistical power. Furthermore, serum IgA rather than intestinal secretory IgA was measured; consequently, the finding does not demonstrate enhanced mucosal IgA secretion, immune exclusion, or intestinal barrier protection.

The negative endpoint associations observed for circulating IL-1β and IL-6 were accompanied by negative associations for the relative expression of *TLR4*, *NFKB1*, *TNF*, *IL1B*, and *IL6* in peripheral blood leukocytes. These broadly corresponding directions are compatible with differences in selected inflammation-related indicators but do not establish attenuation of TLR4/NF-κB signaling. TLR4/NF-κB activity depends on receptor activation and multiple post-transcriptional events, including protein modification, intracellular translocation, and DNA binding, none of which were evaluated in this study [29]. The negative association for *NFKBIA*, which encodes an inhibitor of NF-κB signaling, further precludes interpreting the transcript pattern as uniform suppression of this pathway. In addition, gene expression was measured in mixed leukocyte populations; therefore, the observed patterns may partly reflect differences in leukocyte composition rather than changes in transcription within specific cell populations.

The relative expression of *IL10* and *TGFB1* showed positive linear associations with supplementation level, alongside the positive endpoint association observed for serum IgA. IL-10 and TGF-β participate in context-dependent regulation of inflammatory and immune responses [30]. However, increased transcript abundance does not necessarily indicate increased protein production or biological activity. Circulating IL-10 did not show a statistically significant linear association after FDR correction, and neither circulating TGF-β1 nor functional immune responses were measured. These findings consequently do not establish enhanced immune regulation, immune homeostasis, or a shift toward a regulatory immune balance.

No inflammatory or infectious challenge was applied, and the clinical relevance of the endpoint differences remains uncertain. The findings are therefore most appropriately interpreted as preliminary dose-related associations involving selected circulating and transcriptional immune indicators. They may inform the selection of prospectively designated outcomes and hypotheses for larger confirmatory studies but do not establish improved immune function or a specific immunoregulatory mechanism. Because the tested product was a chemically heterogeneous PKM-derived hydrolysate, the observed associations also cannot be attributed specifically to the identified M2–M6 oligosaccharides.

### 4.3. Ruminal Fermentation

At the single Day 90 sampling conducted 3 h after feeding, propionate and butyrate concentrations showed nominal positive linear associations with the assigned supplementation level, whereas the acetate-to-propionate ratio showed a nominal negative linear association. However, none of these associations remained statistically significant after FDR correction within the ruminal-outcome family (*q* = 0.056 for each outcome). Ruminal pH, acetate concentration, and the sum of the measured acetate, propionate, and butyrate concentrations showed no statistically significant linear or quadratic associations. The findings therefore describe an endpoint pattern in the three quantified VFAs but do not provide statistically robust evidence of a sustained alteration in ruminal fermentation.

The nominal pattern of higher propionate and butyrate concentrations and a lower acetate-to-propionate ratio is compatible with a relatively propionate-oriented profile among the measured VFAs. Propionate is a major glucogenic VFA in ruminants, whereas butyrate is extensively metabolized by the ruminal epithelium and contributes to epithelial energy metabolism and ketone-body formation [31]. Nevertheless, ruminal concentrations measured at one postprandial time point do not directly quantify VFA production, absorption, portal flux, or metabolic utilization. Portal nutrient flux, hepatic glucose production, blood glucose, β-hydroxybutyrate, non-esterified fatty acids, ruminal epithelial morphology, and substrate degradation were not evaluated. The present findings consequently do not demonstrate increased gluconeogenic activity, improved epithelial function, or enhanced systemic energy utilization.

A broadly comparable direction of change in the ruminal VFA profile has been reported in growing beef cattle supplemented with a yeast-derived hydrolysate containing MOS and several other constituents [32]. That study reported increased propionate and butyrate proportions, a reduced acetate-to-propionate ratio, and no detectable improvement in DMI, ADG, or G:F. However, it also reported a quadratic increase in total VFA concentration, which differs from the unchanged sum of the three measured VFAs in the present study. Differences in animal species, basal diet, product composition, dose expression, VFA reporting, and sampling procedures preclude direct comparison. The cattle study therefore provides comparative context for the direction of the VFA profile only and does not establish a common mechanism or an MOS-specific effect.

Ruminal pH ranged from 6.54 to 6.63, with no detectable difference among treatments at the specified sampling time. The absence of a difference in the sum of acetate, propionate, and butyrate suggests that the nominal associations primarily concerned the relative pattern of the three quantified VFAs rather than their combined concentration. Because other VFAs were not quantified, no conclusion can be drawn regarding total ruminal VFA concentration. Furthermore, rumen fluid was collected once, using an oral stomach tube, 3 h after feeding on Day 90. No baseline, serial postprandial, or repeated-day samples were obtained. The measurements therefore represent a single endpoint snapshot and cannot characterize diurnal variation, within-animal changes from baseline, or persistence throughout the 90-day experiment. Oral stomach-tube sampling may also be influenced by salivary contamination and variation in the location of fluid aspiration, although the same collection procedure and timing were applied to all goats [22].

Ruminal microbiota, fermentation kinetics, and substrate degradation were not evaluated. In addition, the tested product was a chemically heterogeneous PKM-derived hydrolysate containing identified M2–M6 oligosaccharides together with residual sugars and other soluble constituents. The nominal endpoint associations cannot therefore be attributed specifically to the identified oligosaccharides, particular microbial taxa, or defined fermentation pathways. Overall, the ruminal findings should be considered preliminary observations concerning selected measures at one postprandial sampling time rather than evidence of a definitive or sustained modification of ruminal fermentation.

### 4.4. Growth Performance and Clinical Tolerance

Final BW, total BW gain, ADG, DMI, and G:F did not differ detectably among supplementation levels. Similar findings have been reported in growing sheep, in which MOS supplementation did not detectably affect feed intake, final BW, or ADG [3]. The goats in the present study were clinically healthy and received a nutritionally adequate basal diet that supported consistent intake and weight gain. Under these non-challenging conditions, the scope for detecting additional improvement in growth performance may have been limited. Nevertheless, the absence of statistically significant differences should not be interpreted as evidence of equivalence because the small sample size limited statistical power and precision.

All goats completely consumed the treatment-containing portion of the ration, and total DMI did not differ detectably among treatments. Thus, supplementation with up to 10.0 g of the PKM-derived hydrolysate/goat/day was not associated with reduced feed acceptance under the conditions of this experiment. All 20 goats completed the 90-day feeding period, and no overt clinical abnormality requiring treatment-related veterinary intervention or withdrawal was observed. These observations support short-term clinical tolerance of the administered amounts but do not establish product safety.

No consistent dose-related pattern was detected across serum total protein, AST, ALT, and ALP. Nominal sampling-time effects were observed for AST and ALP, but these did not remain statistically significant after FDR correction. The measured serum biochemical indices therefore did not provide evidence of a consistent dose-related response. However, the evaluated panel was limited and did not include histopathology, urinalysis, organ-specific biomarkers, oxidative-damage markers, or formal toxicological endpoints. The findings consequently cannot exclude subclinical, organ-specific, uncommon, or longer-term adverse effects.

The hematological findings also require cautious interpretation. Some erythrocyte indices showed nominal or FDR-supported dose-response patterns, but these were not accompanied by a consistent dose-related pattern in RBC count. In addition, the leukocyte differential was obtained using an automated analyzer without confirmatory blood-smear examination. The biological relevance of the erythrocyte-index findings therefore remains uncertain, and they should not be interpreted as evidence of either hematological benefit or toxicity.

Neutrophil percentages were higher, and lymphocyte percentages were lower 4 h after feeding than before feeding, with treatment × sampling-time interactions indicating that the magnitude of these differences varied among treatments. Because these variables were expressed as percentages of the leukocyte differential, reciprocal changes in neutrophil and lymphocyte proportions do not necessarily represent independent changes in absolute cell numbers. Moreover, the contributions of feeding, handling, venipuncture, and normal short-term physiological variation could not be distinguished from treatment-related variation. These observations therefore do not establish treatment-induced inflammation, physiological stress, or an adverse response.

Overall, the absence of reduced feed intake, impaired growth, overt clinical abnormalities, or a consistent adverse pattern across the measured hematological and serum biochemical indices supports preliminary clinical tolerance of the tested supplementation levels during the 90-day experiment. However, the study included only five goats per treatment and was neither designed nor powered as a safety study. Broader toxicological assessment, larger sample sizes, longer observation periods, and confirmatory clinical and pathological measurements are required before the safety of the PKM-derived hydrolysate or a recommended supplementation level can be established.

On the basis of the present evidence, use of the PKM-derived hydrolysate would be most appropriately considered at this stage in controlled research or pilot-feeding settings in which product composition, batch consistency, assigned dose, and individual daily intake can be verified. Its use may be relevant for further investigation when the objective is to examine selected circulating immune indicators or ruminal fermentation measures rather than to improve feed intake or growth performance. The current findings do not support its routine use as a growth-promoting additive or substantiate claims of disease prevention, enhanced immune function, or comprehensive safety. Practical field application should be considered only after an effective and safe supplementation range has been confirmed in larger, adequately powered studies using standardized hydrolysate batches. Such studies should include relevant production settings and, where scientifically and ethically justified, animals exposed to clearly defined nutritional, environmental, or health challenges. Product stability, delivery through practical feeding systems, reproducibility of biological responses, and cost-effectiveness relative to existing feed additives should also be established before routine farm use can be recommended.

### 4.5. Potential Utilization of Palm Kernel Meal

Palm kernel meal is a regionally available co-product that is commonly used as a feed material but is limited by its high fiber content and the restricted accessibility of carbohydrates within its lignocellulosic matrix. Previous research has shown that processed palm kernel cake can be incorporated into goat diets, supporting the regional relevance of palm-derived co-products to small-ruminant production in southern Thailand [33].

In the present study, alkaline pretreatment followed by enzymatic hydrolysis produced a water-soluble, carbohydrate-containing hydrolysate from PKM. Previous studies have likewise demonstrated that PKM or palm kernel cake can be processed to obtain hydrolysates containing mannan-derived sugars and oligosaccharides [6,8,9,10]. However, the present product was chemically heterogeneous. HPLC–RID was used for retention-time-based identification of M2–M6, but the method was not fully validated for quantitative determination. Moreover, the mannan content of the starting PKM was not measured. The present results therefore do not quantify the conversion of PKM mannan into MOS or establish a defined MOS concentration in the recovered product.

Producing a soluble hydrolysate from PKM may represent a potential route for further utilization of this agro-industrial co-product. The reuse of oil-palm biomass and co-products as agricultural inputs has also been discussed within broader circular-bioeconomy frameworks [34]. Nevertheless, the present laboratory-scale experiment did not evaluate complete mass balance, resource and energy consumption, environmental effects, industrial scalability, market value, or economic feasibility. The use of alkaline pretreatment and freeze-drying may be particularly important constraints for practical production.

Accordingly, the findings support further investigation of the PKM-derived hydrolysate as a candidate feed material but do not establish a commercially viable valorization process or demonstrate environmental or circular-bioeconomy benefits. Further work should evaluate compositional consistency across PKM and processing batches, validated quantitative characterization, scalable recovery and drying methods, utilization of the residual solid fraction, product safety and efficacy, and economic and environmental performance. Processing yield and the principal practical constraints are considered further in Section 4.6.

### 4.6. Processing Yield and Practical Feasibility

The laboratory-scale process recovered 12.58 ± 0.42 g of freeze-dried hydrolysate per 100 g of initial dry PKM across three independent processing runs. This value represents the recovery of the complete soluble hydrolysate, including carbohydrates and other soluble constituents, rather than the yield of purified MOS. Peaks corresponding to M2–M6 were identified by retention-time matching with authentic standards using HPLC–RID. However, the chromatographic procedure was not fully validated for quantitative determination. Furthermore, the initial mannan content of the PKM was not measured. Consequently, the concentrations and recovery yields of individual M2–M6 components, MOS purity, mannan-to-MOS conversion efficiency, and complete carbohydrate mass balance could not be established.

The difference between the initial PKM mass and the recovered hydrolysate reflects both the removal of insoluble material during pretreatment and filtration and possible material losses during processing. The present data do not allow these contributions to be distinguished. The procedure also required alkaline pretreatment at 120 °C for 2 h, acid neutralization, enzymatic hydrolysis at 50 °C for 24 h, solid–liquid separation, and freeze-drying. Requirements for alkali, acid, enzyme, water, thermal energy, processing time, and management of liquid and solid residues would therefore need to be considered during scale-up. Freeze-drying was used to obtain a dry laboratory product suitable for chemical characterization and controlled animal supplementation, but its economic suitability for commercial feed production was not evaluated. Process optimization could examine higher-solids operation, reduced chemical and water consumption, optimized enzyme dosage and hydrolysis time, recovery or reuse of process water, utilization of the residual solid fraction, and lower-cost concentration and drying methods such as spray-drying.

No complete process mass balance, production-cost calculation, scale-up study, life-cycle assessment, or techno-economic analysis was conducted. The present results therefore do not demonstrate that the process is commercially viable, environmentally advantageous, or cost-effective relative to commercially available MOS products. Moreover, supplementation did not produce detectable improvements in feed intake or growth performance, and the experiment was not designed to determine an optimal practical dose. Economic evaluation should follow validated quantitative product characterization, improvement of hydrolysate recovery and process consistency, and confirmation of relevant biological responses in larger studies. Such evaluation should consider raw-material variability, product yield and composition, reagent and enzyme requirements, water and energy consumption, residue management, drying and storage costs, product stability, cost per effective animal dose, and comparison with existing feed additives. Accordingly, the present findings provide laboratory-scale evidence to guide further analytical validation and process development but do not support routine farm application or commercial adoption at this stage.

### 4.7. Study Limitations

Several limitations should be considered when interpreting the findings. First, the study included only five goats per treatment, and no formal a priori sample-size calculation was performed for a prospectively designated primary outcome. Although blocking by initial body weight was used to reduce between-animal variability, the limited sample size reduced statistical power and the precision of the estimated treatment effects. Nonsignificant results should therefore not be interpreted as evidence of equivalence or absence of an effect. Conversely, the evaluation of numerous outcomes and contrasts increased the possibility of chance findings. Benjamini–Hochberg false-discovery-rate adjustment was applied within prespecified outcome families, but multiplicity adjustment does not eliminate the uncertainty arising from the small sample size and exploratory design. The findings should consequently be regarded as preliminary and require confirmation in larger, prospectively powered studies with clearly designated primary outcomes.

Second, the experiment was conducted at a single farm using growing male crossbred goats of a similar age. Generalizability to other breeds, sexes, physiological stages, diets, health conditions, and production environments is therefore limited. The supplementation levels were exploratory because no established effective dose of this PKM-derived hydrolysate was available for growing goats. Furthermore, supplementation was administered as fixed amounts per goat per day and was not adjusted as body weight increased. Exposure expressed per kilogram of body weight consequently decreased as the goats grew and may have differed among animals. The observed dose–response patterns apply only to the tested daily amounts under the present experimental conditions and should not be interpreted as defining an optimal, body-weight-normalized, or commercially recommended dose. The absence of a purified or commercial MOS comparator also prevented direct comparison with established products.

Third, Day 0 hematological and serum biochemical measurements were collected for health screening rather than prospectively designated as longitudinal baseline outcomes. These values are presented descriptively in Table S3 but were not incorporated into the Day 90 statistical models. With five goats per treatment, random allocation within initial-body-weight blocks may not have balanced all pretreatment characteristics, and residual baseline differences could have influenced the Day 90 comparisons. Immunoglobulins, cytokines, and immune-related gene expression were measured only in prefeeding samples collected on Day 90; corresponding pretreatment values were unavailable. The observed immune-related patterns therefore represent between-group endpoint differences and cannot establish treatment-induced within-animal changes. Similarly, hematological and serum biochemical measurements obtained before feeding and 4 h after feeding on Day 90 describe values at two sampling times but do not characterize complete postprandial or longitudinal responses.

Fourth, the experiment was conducted without blinding. Personnel involved in supplement administration, animal monitoring, sample collection, laboratory analyses, data management, and statistical analysis were aware of treatment assignments. Standardized management, sampling, processing, instrument settings, assay procedures, outcome definitions, and statistical models were applied across treatment groups. Nevertheless, these measures cannot eliminate observer, detection, or analytical bias associated with the open-label design, particularly for procedures involving manual sample handling or analytical decisions.

Fifth, several analytical limitations should be acknowledged. The tested product was a chemically heterogeneous PKM-derived hydrolysate rather than purified MOS. M2–M6 were identified by HPLC–RID retention-time matching with authentic standards, but the chromatographic procedure was not fully validated for quantitative determination, and compound identity was not confirmed by mass spectrometry. Possible co-elution with other carbohydrates cannot be excluded. Because the initial mannan content of the PKM was also not measured, MOS purity, recovery, and mannan-to-MOS conversion efficiency could not be established. Other carbohydrates, protein, ash, and unidentified soluble constituents may have contributed to the observed responses. In addition, the ELISA kits were not independently validated for the goat serum matrix through dilutional parallelism, spike-and-recovery testing, or formal assessment of matrix effects. Manufacturer-reported assay characteristics should therefore not be interpreted as in-house validation, and the immunoglobulin and cytokine findings require confirmation using independently validated assays.

RNA integrity was not evaluated by gel electrophoresis or an automated integrity platform; therefore, RNA integrity numbers were unavailable. The A260/A280 ratio provided an indicator of spectrophotometric purity but not RNA degradation. Moreover, gene expression was measured in mixed peripheral blood leukocytes rather than purified leukocyte subpopulations. Variation in leukocyte composition may therefore have contributed to the observed expression patterns, and GAPDH normalization does not correct for differences in the proportions of leukocyte subsets. The results cannot consequently be attributed unequivocally to transcriptional regulation within a specific leukocyte population.

Finally, ruminal pH and acetate, propionate, and butyrate concentrations were determined from a single oral stomach-tube sample collected 3 h after feeding on Day 90. The absence of pretreatment, serial postprandial, and repeated-day sampling prevented assessment of within-animal changes, diurnal variation, and persistence of the observed differences. Oral stomach-tube sampling may also be influenced by salivary contamination and variation in the ruminal location of fluid aspiration. Ruminal microbiota, fermentation kinetics, substrate degradation, intestinal or ruminal tissue morphology, and barrier function were not evaluated. Blood glucose, β-hydroxybutyrate, non-esterified fatty acids, VFA absorption, portal nutrient flux, and hepatic glucose production were also not measured. The ruminal results therefore cannot establish a sustained fermentation response, a specific microbial mechanism, increased gluconeogenesis, or improved energy metabolism.

The goats were clinically healthy and were not subjected to an infectious or inflammatory challenge. The clinical relevance of the observed immune-related endpoint differences and their applicability under disease or stress conditions remain uncertain. In addition, the limited clinical observations and measured hematological and serum biochemical variables were insufficient to establish comprehensive product safety. The hydrolysate was produced from a single PKM batch at laboratory scale, and its batch consistency, storage stability, industrial scalability, environmental performance, and economic feasibility were not established. Collectively, these limitations indicate that the findings should be interpreted as preliminary associations involving the complete PKM-derived hydrolysate rather than definitive evidence of MOS-specific efficacy, safety, mechanism of action, or practical applicability.

## 5. Conclusions

In this preliminary study of five goats per treatment, graded supplementation with a PKM-derived MOS-containing hydrolysate was associated with higher Day 90 serum IgA and lower IL-1β and IL-6 concentrations, together with differences in selected immune-related transcripts; these associations remained significant after FDR correction. Nominal associations with propionate, butyrate, and the acetate-to-propionate ratio at a single Day 90 postprandial sampling did not remain significant after FDR correction. Feed intake and growth performance did not differ detectably among treatments. Because of the small sample size, absence of pretreatment immune and ruminal measurements, open-label design, limited assay validation, and chemically heterogeneous composition of the hydrolysate, these findings do not establish efficacy, safety, a specific mechanism, or a recommended dose. Confirmation in larger, adequately powered studies with prospectively designated primary outcomes and repeated measurements is required. At present, the hydrolysate is most appropriately regarded as a candidate ingredient for controlled research or pilot-feeding evaluation rather than a supplement recommended for routine growth promotion or farm application.

## Figures and Tables

**Figure 1:**
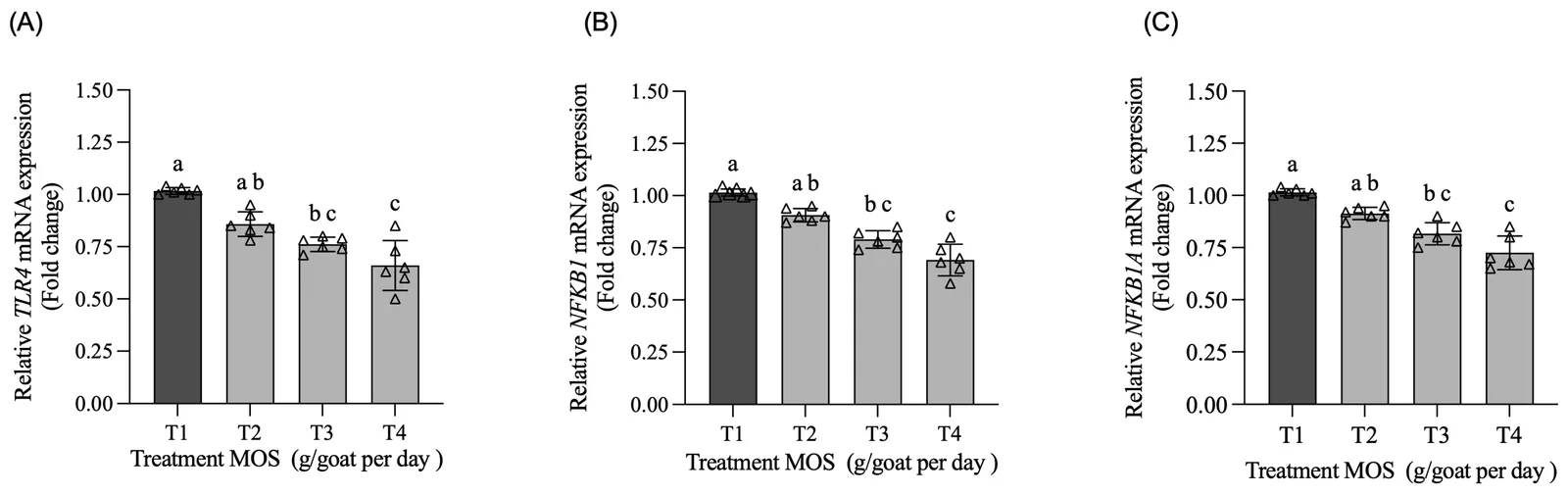
Effects of graded supplementation with the PKM-derived MOS-rich hydrolysate on innate immune recognition and NF-κB-related signaling in peripheral blood leukocytes of growing goats. Relative mRNA expression of (**A**) *TLR4*, (**B**) *NFKB1*, and (**C**) *NFKBIA*. T1 = CON, T2 = MOS2.5, T3 = MOS5, and T4 = MOS10 supplied 0, 2.5, 5.0, and 10.0 g hydrolysate/goat/day, respectively. Gene expression was normalized to *GAPDH* and calculated using the 2^−ΔΔCq^ method, with CON used as the calibrator. Individual symbols represent biologically independent animals, and bars and error bars represent the mean ± SD (*n* = 5 animals/treatment). Means without a common letter differ according to Tukey-adjusted pairwise comparisons (*p* < 0.05).

**Figure 2:**
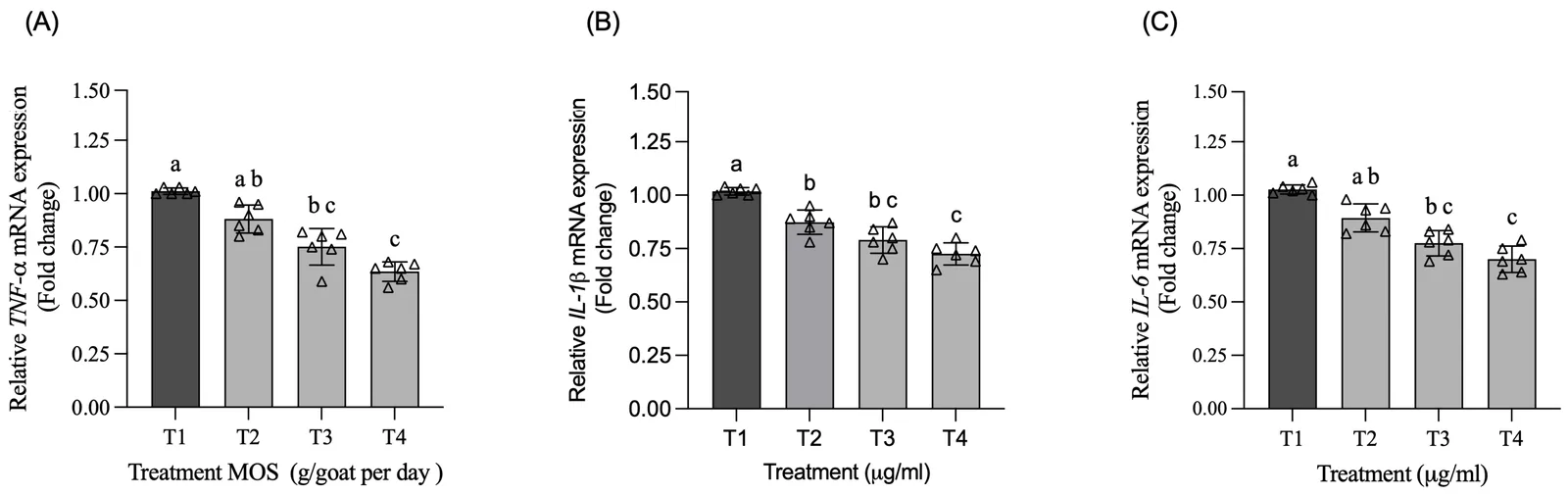
Effects of graded supplementation with the PKM-derived MOS-rich hydrolysate on pro-inflammatory gene expression in peripheral blood leukocytes of growing goats. Relative mRNA expression of (**A**) *TNFA*, (**B**) *IL1B*, and (**C**) *IL6*. T1 = CON, T2 = MOS2.5, T3 = MOS5, and T4 = MOS10 supplied 0, 2.5, 5.0, and 10.0 g hydrolysate/goat/day, respectively. Gene expression was normalized to *GAPDH* and calculated using the 2^−ΔΔCq^ method, with CON used as the calibrator. Individual symbols represent biologically independent animals, and bars and error bars represent the mean ± SD (*n* = 5 animals/treatment). Means without a common letter differ according to Tukey-adjusted pairwise comparisons (*p* < 0.05).

**Figure 3:**
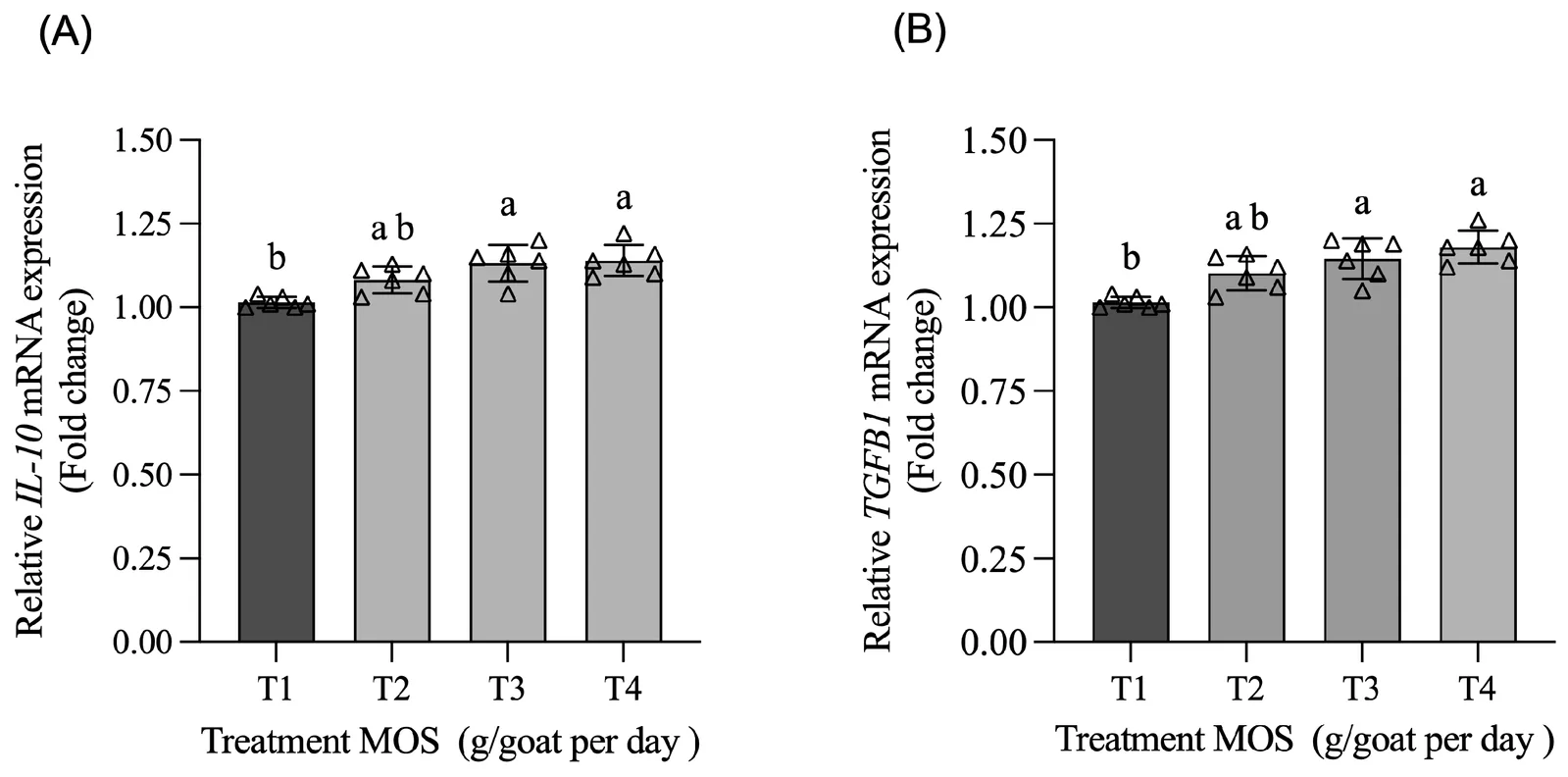
Effects of graded supplementation with the PKM-derived MOS-rich hydrolysate on regulatory gene expression in peripheral blood leukocytes of growing goats. Relative mRNA expression of (**A**) *IL10* and (**B**) *TGFB1*. T1 = CON, T2 = MOS2.5, T3 = MOS5, and T4 = MOS10 supplied 0, 2.5, 5.0, and 10.0 g hydrolysate/goat/day, respectively. Treatment definitions, gene-expression normalization, calculations, and graphical presentation are as described for Figure 2. Individual symbols represent biologically independent animals, and bars and error bars represent the mean ± SD (*n* = 5 animals/treatment). Means without a common letter differ according to Tukey-adjusted pairwise comparisons (*p* < 0.05).

**Table 1:** Ingredient composition and calculated chemical composition of the basal total mixed ration fed to growing goats.

Item	TMR
Ingredient composition (% DM)	
Pangola grass hay	30.00
Ground corn	35.80
Soybean meal	23.10
Fish meal	0.60
Leucaena leaf meal	4.20
Molasses	4.70
Dicalcium phosphate	0.35
Salt	0.25
Vitamin–mineral premix ^1^	1.00
Total	100.00
Chemical composition	
Dry matter (% as-fed)	90.00
Crude protein (% DM)	16.46
Ash (% DM)	5.90
Organic matter (% DM)	94.10
Ether extract (% DM)	2.88
Neutral detergent fiber (% DM)	52.23
Acid detergent fiber (% DM)	26.43
Acid detergent lignin (% DM)	5.76
Non-fibrous carbohydrate (% DM) ^2^	22.53
Total digestible nutrients (% DM)	69.55
Metabolizable energy (Mcal/kg DM)	2.50

^1^ The vitamin–mineral premix provided per kilogram of premix: vitamin A, 10,000,000 IU; vitamin D_3_, 1,600,000 IU; vitamin E, 70,000 IU; iron, 50 g; zinc, 40 g; manganese, 40 g; copper, 10 g; iodine, 0.5 g; cobalt, 0.1 g; and selenium, 0.1 g. ^2^ Non-fibrous carbohydrate was calculated as 100 − (crude protein + neutral detergent fiber + ether extract + ash), with all components expressed as percentages of dry matter. Except for dry matter, the chemical values were calculated from the analyzed composition of the concentrate mixture and Pangola grass hay, weighted at 70% and 30% of dietary dry matter, respectively. Total digestible nutrients and metabolizable energy were estimated as described in Section 2.7. DM, dry matter; TMR, total mixed ration.

**Table 2:** Yield and physicochemical characteristics of the PKM-derived hydrolysate.

Parameter	Value
Hydrolysate yield (% of initial PKM dry matter)	12.58 ± 0.42
Moisture content (% as-is)	4.82 ± 0.21
Total carbohydrate (% DM)	72.46 ± 1.83
Reducing sugars (% DM)	58.27 ± 1.46
Crude protein (% DM)	6.35 ± 0.32
Ash (% DM)	5.18 ± 0.27
Other components (% DM) ^1^	16.01 ± 1.88
Water solubility (% of initial dry sample weight)	93.64 ± 1.12
pH of a 1% (*w*/*v*) solution	5.62 ± 0.08

Values are presented as the mean ± standard deviation of three independent processing runs. Each processing replicate was analyzed in triplicate. Moisture content is reported on an as-is basis; total carbohydrate, reducing sugars, crude protein, ash, and other components are reported on a dry matter basis. Reducing sugars are included within the total carbohydrate fraction and should not be added separately when calculating total composition. DM, dry matter. ^1^ The unaccounted fraction was calculated separately for each processing run as 100 minus the sum of total carbohydrate, crude protein, and ash, with all components expressed on a dry matter basis.

**Table 3:** HPLC–RID identification and quantitative composition of manno-oligosaccharides in the PKM-derived hydrolysate.

Compound	Standard RT (min)	Sample RT (min)	Content (mg/g Hydrolysate)	Content (% *w*/*w*)	Proportion of Total MOS (%)
Mannobiose (M2)	8.50	8.52	205.0 ± 4.1	20.50 ± 0.41	38.35
Mannotriose (M3)	10.00	10.03	145.0 ± 3.8	14.50 ± 0.38	27.12
Mannotetraose (M4)	11.20	11.23	95.0 ± 3.5	9.50 ± 0.35	17.77
Mannopentaose (M5)	12.00	12.04	55.0 ± 4.0	5.50 ± 0.40	10.29
Mannohexaose (M6)	12.85	12.89	34.6 ± 3.7	3.46 ± 0.37	6.47
Total MOS (M2–M6)	—	—	534.6 ± 8.6	53.46 ± 0.86	100.00

Values are presented as the mean ± SD of three independent hydrolysate production runs, each analyzed in triplicate. RT, retention time; MOS, manno-oligosaccharides. Compounds were assigned by comparing their retention times with those of authentic standards analyzed under identical chromatographic conditions. The proportion of total MOS was calculated relative to the summed concentrations of M2–M6.

**Table 4:** Effects of graded supplementation with the PKM-derived MOS-rich hydrolysate on feed intake and growth performance in growing goats.

Parameter	CON, LSM (95% CI)	MOS2.5, LSM (95% CI)	MOS5, LSM (95% CI)	MOS10, LSM (95% CI)	SEM	Linear *p*-Value	Quadratic *p*-Value
Initial BW, kg ^1^	17.46 ± 3.70	17.54 ± 3.21	17.48 ± 3.09	17.52 ± 3.47	-	-	-
Final BW, kg	30.3 (28.10–32.50)	30.4 (28.20–32.60)	31.2 (29.00–33.40)	31.6 (29.40–33.80)	1.12	0.362	0.895
BW gain, kg	12.8 (10.57–15.03)	12.9 (10.67–15.13)	13.7 (11.47–15.93)	14.1 (11.87–16.33)	1.14	0.370	0.897
ADG, kg/day	0.142 (0.117–0.167)	0.143 (0.118–0.168)	0.152 (0.127–0.177)	0.157 (0.132–0.182)	0.013	0.367	0.880
ADG, g/kg BW^0.75^/day	13.04 (11.20–14.88)	12.96 (11.12–14.80)	14.12 (12.28–15.96)	14.15 (12.31–15.99)	0.94	0.301	0.954
DMI, kg/day	0.723 (0.645–0.801)	0.715 (0.637–0.793)	0.764 (0.686–0.842)	0.768 (0.690–0.846)	0.040	0.319	0.883
DMI, % BW	3.03 (2.91–3.15)	2.99 (2.87–3.11)	3.14 (3.02–3.26)	3.13 (3.01–3.25)	0.06	0.113	0.806
DMI, g/kg BW^0.75^/day	68.3 (64.52–72.08)	66.6 (62.82–70.38)	69.1 (65.32–72.88)	69.8 (66.02–73.58)	1.93	0.431	0.545
OM intake, kg/day	0.683 (0.624–0.742)	0.650 (0.591–0.709)	0.725 (0.666–0.784)	0.723 (0.664–0.782)	0.030	0.165	0.612
CP intake, kg/day	0.115 (0.095–0.135)	0.113 (0.093–0.133)	0.123 (0.103–0.143)	0.124 (0.104–0.144)	0.010	0.420	0.883
NDF intake, kg/day	0.335 (0.315–0.355)	0.334 (0.314–0.354)	0.357 (0.337–0.377)	0.358 (0.338–0.378)	0.010	0.056	0.922
ADF intake, kg/day	0.163 (0.143–0.183)	0.156 (0.136–0.176)	0.163 (0.143–0.183)	0.164 (0.144–0.184)	0.010	0.826	0.694
G:F, kg gain/kg DMI	0.196 (0.176–0.216)	0.200 (0.180–0.220)	0.199 (0.179–0.219)	0.204 (0.184–0.224)	0.010	0.614	0.961

Values other than initial BW are least-squares means, with five goats per treatment. SEM represents the standard error of the model-estimated treatment means. ^1^ Initial BW values are unadjusted arithmetic means ± SD calculated from the individual Day 0 measurements. Initial BW was used to form the five body-weight blocks before random treatment allocation and was therefore not subjected to linear or quadratic dose-response testing. Each individually housed and individually fed goat was considered one experimental unit. Linear and quadratic contrasts were constructed using the assigned supplementation levels of 0, 2.5, 5.0, and 10.0 g/goat/day. Benjamini–Hochberg false-discovery-rate adjustment was applied separately to the linear and quadratic contrasts within the growth-and-intake outcome family. Both unadjusted *p*-values and FDR-adjusted *q*-values are reported; *q* < 0.05 was considered statistically significant after multiplicity correction. BW, body weight; ADG, average daily gain; DMI, dry-matter intake; OM, organic matter; CP, crude protein; NDF, neutral detergent fiber; ADF, acid detergent fiber; G:F, gain-to-feed ratio; SEM, standard error of the mean.

**Table 5:** Effects of graded supplementation with the PKM-derived MOS-rich hydrolysate on hematological indices in growing goats.

Parameter	Sampling Time	CON, LSM (95% CI)	MOS2.5, LSM (95% CI)	MOS5, LSM (95% CI)	MOS10, LSM (95% CI)	SEM	Linear *p*	Quadratic *p*	Time *p*	Treatment × Time *p*
RBC, ×10^6^/µL	Overall	14.16 (13.69–14.63)	13.28 (12.81–13.75)	14.44 (13.97–14.91)	14.52 (14.05–14.99)	0.24	0.100	0.100	0.170	0.080
MCV, fL	Overall	21.89 (21.60–22.18)	23.48 (23.19–23.77)	19.69 (19.40–19.98)	20.15 (19.86–20.44)	0.15	<0.001	0.310	0.620	0.490
MCH, pg	0 h	7.54 (7.29–7.79)	8.63 (8.38–8.88)	7.71 (7.46–7.96)	7.33 (7.08–7.58)	0.13	0.080	<0.001	0.080	0.030
	4 h	8.14 (7.90–8.38)	8.50 (8.26–8.74)	7.50 (7.26–7.74)	7.65 (7.41–7.89)	0.12	0.010	0.550	—	—
MCHC, g/dL	Overall	35.92 (35.49–36.35)	36.08 (35.65–36.51)	37.21 (36.78–37.64)	36.01 (35.58–36.44)	0.22	0.290	0.030	0.020	0.620
WBC, ×10^3^/µL	Overall	15.56 (14.80–16.32)	17.22 (16.46–17.98)	13.57 (12.81–14.33)	15.59 (14.83–16.35)	0.39	0.270	0.800	0.480	0.580
Neutrophils, %	0 h	34.52 (31.83–37.21)	29.61 (26.92–32.30)	30.26 (27.57–32.95)	39.28 (36.59–41.97)	1.37	0.020	<0.001	<0.001	0.003
	4 h	59.40 (55.97–62.83)	48.38 (44.95–51.81)	52.33 (48.90–55.76)	49.02 (45.59–52.45)	1.75	0.010	0.080	—	—
Lymphocytes, %	0 h	65.85 (62.69–69.01)	56.33 (53.17–59.49)	61.87 (58.71–65.03)	53.98 (50.82–57.14)	1.61	0.010	0.720	<0.001	<0.001
	4 h	33.55 (28.69–38.41)	46.49 (41.63–51.35)	41.29 (36.43–46.15)	39.28 (34.42–44.14)	2.48	0.330	0.010	—	—
Monocytes, %	Overall	2.51 (2.14–2.88)	2.55 (2.18–2.92)	2.23 (1.86–2.60)	2.13 (1.76–2.50)	0.19	0.090	0.700	<0.001	0.390

Unless otherwise indicated, values are estimated marginal means (least-squares means) averaged across the two Day 90 sampling times, based on five goats per treatment. For outcomes with a treatment × sampling-time interaction at raw *p* < 0.05, treatment means and dose-response contrasts are presented separately at 0 h and 4 h after feeding. The 0-h samples were collected immediately before the morning feeding. SEM denotes the standard error of the model-estimated means. CON, MOS2.5, MOS5, and MOS10 supplied 0, 2.5, 5.0, and 10.0 g of the PKM-derived hydrolysate/goat/day, respectively. Linear and quadratic contrasts were constructed using the assigned supplementation levels. Sampling-time and treatment × sampling-time *p*-values are reported once for each outcome because they were obtained from the overall repeated-measures mixed-effects model. The corresponding FDR-adjusted *q*-values are provided in Supplementary Table S4. Neutrophil and lymphocyte values represent percentages of the leukocyte differential rather than absolute cell counts. RBC, red blood cell count; MCV, mean corpuscular volume; MCH, mean corpuscular hemoglobin; MCHC, mean corpuscular hemoglobin concentration; WBC, white blood cell count; SEM, standard error of the mean.

**Table 6:** Effects of graded supplementation with the PKM-derived MOS-rich hydrolysate on serum biochemical indices in growing goats.

Parameter	CON, LSM (95% CI)	MOS2.5, LSM (95% CI)	MOS5, LSM (95% CI)	MOS10, LSM (95% CI)	SEM	Linear *p*	Quadratic *p*	Time *p*	Treatment × Time *p*
Total protein, g/dL	6.02 (5.51–6.53)	6.21 (5.70–6.72)	6.24 (5.73–6.75)	6.27 (5.76–6.78)	0.26	0.640	0.760	0.860	0.510
AST, U/L	108.06 (97.59–118.53)	90.44 (79.97–100.91)	109.00 (98.53–119.47)	107.69 (97.22–118.16)	5.34	0.550	0.260	0.040	0.380
ALT, U/L	26.86 (23.74–29.98)	23.46 (20.34–26.58)	21.17 (18.05–24.29)	23.12 (20.00–26.24)	1.59	0.290	0.390	0.720	0.360
ALP, U/L	419.89 (353.25–486.53)	327.08 (260.44–393.72)	248.03 (181.39–314.67)	340.45 (273.81–407.09)	34.00	0.120	0.520	0.020	0.440

Values are least-squares means across the two sampling times (*n* = 5 goats/treatment). CON, MOS2.5, MOS5, and MOS10 supplied 0, 2.5, 5.0, and 10.0 g hydrolysate/goat/day, respectively. AST, aspartate aminotransferase; ALT, alanine aminotransferase; ALP, alkaline phosphatase; SEM, standard error of the mean.

**Table 7:** Circulating immunoglobulin and cytokine concentrations measured at the Day 90 endpoint in growing goats receiving graded supplementation with the PKM-derived MOS-containing hydrolysate.

Parameter	CON, LSM (95% CI)	MOS2.5, LSM (95% CI)	MOS5, LSM (95% CI)	MOS10, LSM (95% CI)	SEM	Linear *p*	Linear *q*	Quadratic *p*	Quadratic *q*
Immunoglobulins									
IgA, µg/mL	46.24 (44.91–47.57)	47.31 (45.98–48.64)	48.62 (47.29–49.95)	50.01 (48.68–51.34)	0.68	0.018	0.036	0.790	0.790
IgG, mg/mL	20.12 (19.57–20.67)	20.74 (20.19–21.29)	21.28 (20.73–21.83)	21.16 (20.61–21.71)	0.28	0.170	0.204	0.520	0.780
IgM, mg/mL	1.78 (1.49–2.07)	1.77 (1.48–2.06)	1.82 (1.53–2.11)	1.81 (1.52–2.10)	0.15	0.440	0.440	0.770	0.790
Cytokines									
IL-1β, pg/mL	218.37 ^a^ (210.88–225.86)	201.52 ^b^ (194.03–209.01)	191.84 ^b^ (184.35–199.33)	186.45 ^b^ (178.96–193.94)	3.82	0.003	0.012	0.090	0.340
IL-6, pg/mL	145.62 ^a^ (140.76–150.48)	137.84 ^ab^ (132.98–142.70)	129.96 ^b^ (125.10–134.82)	126.73 ^b^ (121.87–131.59)	2.48	0.004	0.012	0.170	0.340
IL-10, pg/mL	241.83 (234.09–249.57)	249.46 (241.72–257.20)	264.71 (256.97–272.45)	261.35 (253.61–269.09)	3.95	0.061	0.091	0.120	0.340

Values are model-estimated least-squares means measured in prefeeding serum samples collected on Day 90, with five goats per treatment. Immunoglobulins and cytokines were not measured before supplementation; therefore, these results represent between-group endpoint comparisons rather than within-animal changes from baseline. Linear and quadratic contrasts were constructed using the assigned supplementation levels of 0, 2.5, 5.0, and 10.0 g/goat/day. Benjamini–Hochberg false-discovery-rate adjustment was applied across the six circulating immune indicators separately for the linear and quadratic contrasts. Both unadjusted *p*-values and FDR-adjusted *q*-values are reported; *q* < 0.05 was considered statistically significant after multiplicity correction. Within a row, means without a common superscript differ according to Tukey-adjusted pairwise comparisons (*p* < 0.05). Ig, immunoglobulin; IL, interleukin; SEM, standard error of the mean.

**Table 8:** Effects of graded supplementation with the PKM-derived MOS-rich hydrolysate on ruminal pH and volatile fatty acid profiles in growing goats.

Parameter	CON, LSM (95% CI)	MOS2.5, LSM (95% CI)	MOS5, LSM (95% CI)	MOS10, LSM (95% CI)	SEM	Linear *p*	Quadratic *p*
Ruminal pH	6.63 (6.55–6.71)	6.56 (6.48–6.64)	6.58 (6.50–6.66)	6.54 (6.46–6.62)	0.041	0.284	0.721
Acetate, mmol/L	62.1 (59.59–64.61)	63.8 (61.29–66.31)	63.2 (60.69–65.71)	64.5 (61.99–67.01)	1.28	0.608	0.846
Propionate, mmol/L	20.8 ^b^ (19.45–22.15)	23.5 ^a^ (22.15–24.85)	22.3 ^ab^ (20.95–23.65)	23.1 ^ab^ (21.75–24.45)	0.69	0.028	0.164
Butyrate, mmol/L	10.4 ^b^ (9.69–11.11)	11.3 ^ab^ (10.59–12.01)	11.0 ^ab^ (10.29–11.71)	11.8 ^a^ (11.09–12.51)	0.36	0.021	0.081
The sum of measured VFAs, mmol/L ^1^	93.3 (89.36–97.24)	98.6 (94.66–102.54)	96.5 (92.56–100.44)	99.4 (95.46–103.34)	2.01	0.118	0.687
Acetate-to-propionate ratio	2.99 ^a^ (2.85–3.13)	2.72 ^b^ (2.58–2.86)	2.83 ^ab^ (2.69–2.97)	2.79 ^ab^ (2.65–2.93)	0.07	0.018	0.237

Values are least-squares means (*n* = 5 goats/treatment). CON, MOS2.5, MOS5, and MOS10 supplied 0, 2.5, 5.0, and 10.0 g hydrolysate/goat/day, respectively. Linear and quadratic contrasts were constructed using the actual supplementation levels. Within a row, means without a common superscript differ according to Tukey-adjusted pairwise comparisons (*p* < 0.05). A tendency was defined as 0.05 ≤ *p* < 0.10. SEM, standard error of the mean. VFA, volatile fatty acids. ^1^ The sum of measured VFAs represents the combined concentrations of acetate, propionate, and butyrate; other VFAs were not quantified.

## Data Availability

The minimal dataset supporting the findings of this study is provided in the Supplementary Materials. Additional de-identified data and analytical outputs are available from the corresponding author upon reasonable request.

## Supplementary Materials

The following supporting information can be downloaded at https://www.mdpi.com/article/10.3390/vetsci13101008/s1, Figure S1: HPLC–RID chromatograms of authentic carbohydrate standards and the PKM-derived MOS-rich hydrolysate; Table S1: Primer sequences and amplification characteristics used for one-step RT-qPCR analysis of immune-related gene expression in peripheral blood leukocytes of growing goats [35,36,37]; Table S2: Complete statistical results for immune-related gene expression in peripheral blood leukocytes of growing goats; Table S3: Baseline hematological and serum biochemical values measured before the initiation of supplementation on Day 0; Table S4: Hematological indices measured before and 4 h after feeding; Table S5: Serum biochemical indices measured before and 4 h after feeding.

## Abbreviations

The following abbreviations are used in this manuscript:

MOS	Mannan-Oligosaccharides
PKM	Palm Kernel Meal
IgA	Immunoglobulin A
VFA/VFAs	Volatile Fatty Acid/Volatile Fatty Acids
HPLC-RID	High-Performance Liquid Chromatography with Refractive Index Detection
TMR	Total Mixed Ration
DM	Dry Matter
CP	Crude Protein
EE	Ether Extract
NDF	Neutral Detergent Fiber
ADF	Acid Detergent Fiber
DMI	Dry Matter Intake
BW	Body Weight
ADG	Average Daily Gain
G:F	Gain-to-Feed Ratio
PBL/PBLs	Peripheral Blood Leukocytes
RBC	Red Blood Cell (Erythrocyte Count)
WBC	White Blood Cell (Leukocyte Count)
ELISA	Enzyme-Linked Immunosorbent Assay
IgG	Immunoglobulin G
IgM	Immunoglobulin M
RT-qPCR	Reverse Transcription Quantitative Polymerase Chain Reaction
GC-FID	Gas Chromatography with Flame Ionization Detection
FDR	False Discovery Rate
A:P ratio	Acetate-to-Propionate ratio
