# Supplementary material for: Dose-Related Associations of a Palm Kernel Meal-Derived Mannan-Oligosaccharide-Containing Hydrolysate with Selected Immune and Ruminal Fermentation Measures in Growing Goats
Source: Vet Sci. 2026 Sep 24;13(10):1008. doi: 10.3390/vetsci13101008 (PMC13709411; doi:10.3390/vetsci13101008)
Supplement: Supplementary file 1 [file vetsci-13-01008-s001.zip › vetsci-4543207-supplementary.pdf]

Supplementary Materials

Figure S1. HPLC–RID chromatograms of authentic carbohydrate standards and the PKM-derived MOS-rich hydrolysate.

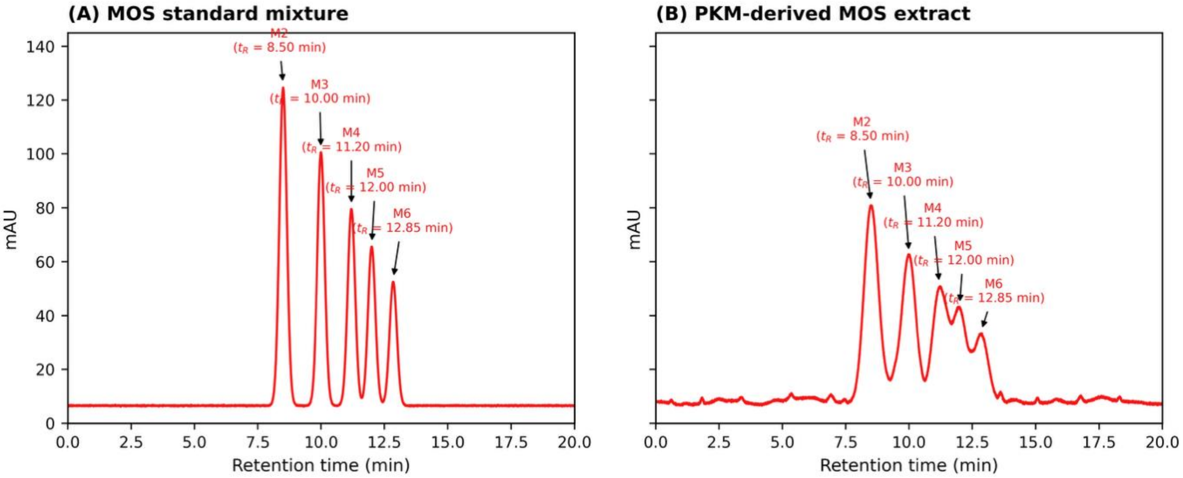

Analyte	Calibration range (mg/mL)	Number of calibration levels	Regression model	R^2
Mannobiose (M2)	0.025–1.000	6	Linear, peak area versus concentration	0.9989
Mannotriose (M3)	0.025–1.000	6	Linear, peak area versus concentration	0.9984
Mannotetraose (M4)	0.025–1.000	6	Linear, peak area versus concentration	0.9978
Mannopentaose (M5)	0.025–1.000	6	Linear, peak area versus concentration	0.9972
Mannohexaose (M6)	0.025–1.000	6	Linear, peak area versus concentration	0.9968

Calibration standards were prepared at 0.025, 0.050, 0.100, 0.250, 0.500, and 1.000 mg/mL. Calibration curves were constructed by plotting HPLC–RID peak area against analyte concentration and fitting an unweighted linear regression model. The calibration characteristics were evaluated solely to support fit-for-purpose external-standard quantification of M2–M6 in the PKM-derived hydrolysate. The procedure was not subjected to full analytical-method validation; therefore, method-validation parameters such as the limit of detection, limit of quantification, accuracy, recovery, and intra- or inter-day precision are not reported.

Table S1. Primer sequences and amplification characteristics used for one-step RT-qPCR analysis of immune-related gene expression in peripheral blood leukocytes of growing goats

Gene	Accession number	Forward primer (5′–3′)	Reverse primer (5′–3′)	Amplicon size (bp)	Efficiency (%)	Reference
GAPDH	XM_005680968.3	CGGCACAGTCAAGGCAGAGAAC	CACGTACTCAGCACCAGCATCAC	115	98.2	[35]
TLR4	NM_001285574.1	TTCGCATCTGGATAAATCCAGC	CTGAGAACCGAGAGCTGGGAC	207	96.7	[35]
NFKB1	XM_018043384.1	CTCACCAATGGCCTCCTCTC	ACACCCTCCCAGAATCCGTA	179	97.4	[35]
NFKBIA	XM_013973127	GTTGAAGTGTGGGCTGATG	TCATCGTAGGGAAGCTCGTC	173	95.8	[36]
TNF (*TNFA*)	XM_005696606.3	CAAGTAACAAGCCGGTAGCC	AGATGAGGTAAAGCCCGTCA	153	99.1	[35]
IL1B	XM_013967700.2	CATGTGTGCTGAAGGCTCTC	AGTGTCGGCGTATCACCTTT	172	94.9	[35]
IL6	NM_001285640.1	CCAATCTGGGTTCAATCAGG	ACCCACTCGTTTGAGGACTG	240	96.2	[35]
IL10	XM_005690416.3	TTAAGGGTTACCTGGGTTGC	CCCTCTCTTGGAGCATATTGA	237	97.7	[35]
TGFB1	NM_001314142.1	CAACAATTCCTGGCGCTACCT	ATGTCCACTTGAAGCGTGTTATCC	183	95.5	[37]

Table S2. Complete statistical results for immune-related gene expression in peripheral blood leukocytes of growing goats.

Gene	CON	MOS2.5	MOS5	MOS10	Linear raw *p*	Linear *q*	Quadratic raw *p*	Quadratic *q*
TLR4	1.00 ± 0.01[a]	0.86 ± 0.03[ab]	0.76 ± 0.02[bc]	0.66 ± 0.05[c]	0.002	0.004	0.221	0.315
NFKB1	1.00 ± 0.01[a]	0.90 ± 0.03[ab]	0.79 ± 0.04[bc]	0.70 ± 0.04[c]	0.003	0.005	0.259	0.315
NFKBIA	1.00 ± 0.01[a]	0.90 ± 0.04[ab]	0.80 ± 0.04[bc]	0.71 ± 0.04[c]	0.004	0.005	0.287	0.315
TNF (*TNFA*)	1.00 ± 0.01[a]	0.87 ± 0.03[ab]	0.75 ± 0.04[bc]	0.64 ± 0.03[c]	0.001	0.004	0.146	0.315
IL1B	1.00 ± 0.01[a]	0.88 ± 0.03[b]	0.78 ± 0.03[bc]	0.72 ± 0.03[c]	0.001	0.004	0.188	0.315
IL6	1.02 ± 0.02[a]	0.90 ± 0.04[ab]	0.76 ± 0.04[bc]	0.69 ± 0.04[c]	0.002	0.004	0.315	0.315
IL10	1.00 ± 0.01[b]	1.07 ± 0.03[ab]	1.13 ± 0.03[a]	1.12 ± 0.03[a]	0.008	0.008	0.064	0.315
TGFB1	1.00 ± 0.01[b]	1.09 ± 0.04[ab]	1.14 ± 0.03[a]	1.17 ± 0.04[a]	0.005	0.006	0.119	0.315

Table S2—continued

Tukey-adjusted pairwise comparisons for immune-related gene expression.

Gene	CON vs. MOS2.5	CON vs. MOS5	CON vs. MOS10	MOS2.5 vs. MOS5	MOS2.5 vs. MOS10	MOS5 vs. MOS10
TLR4	0.082	0.012	0.001	0.164	0.019	0.211
NFKB1	0.116	0.018	0.002	0.193	0.027	0.246
NFKBIA	0.137	0.024	0.003	0.218	0.034	0.271
TNF (*TNFA*)	0.071	0.009	<0.001	0.142	0.012	0.184
IL1B	0.041	0.006	<0.001	0.151	0.018	0.328
IL6	0.094	0.011	0.001	0.128	0.021	0.263
IL10	0.184	0.021	0.029	0.316	0.382	0.997
TGFB1	0.143	0.032	0.009	0.487	0.164	0.611

Values are estimated marginal means ± SEM (n = 5 goats/treatment). Gene expression was normalized to *GAPDH* and calculated using the method, with the CON group used as the calibrator. CON, MOS2.5, MOS5, and MOS10 supplied 0, 2.5, 5.0, and 10.0 g hydrolysate/goat/day, respectively. Linear and quadratic contrasts were constructed using the actual supplementation levels. Raw contrast *p*-values were adjusted across the eight immune-related genes using the Benjamini–Hochberg false-discovery-rate procedure. Within a row, means without a common superscript differ according to Tukey-adjusted pairwise comparisons ($p < 0.05$).

Table S3. Baseline hematological and serum biochemical values measured before the initiation of supplementation on Day 0.

Parameter	CON (n = 5)	MOS2.5 (n = 5)	MOS5 (n = 5)	MOS10 (n = 5)
RBC, $\times 10^6$/μL	17.03 ± 3.34	17.55 ± 1.61	17.53 ± 2.37	16.27 ± 1.37
MCV, fL	16.28 ± 0.70	16.78 ± 0.68	14.60 ± 1.58	15.44 ± 1.02
MCH, pg	6.72 ± 0.42	6.92 ± 0.23	6.16 ± 0.37	6.36 ± 0.35
MCHC, g/dL	41.64 ± 1.07	41.60 ± 1.31	42.80 ± 2.29	41.50 ± 1.11
WBC, $\times 10^3$/μL	17.08 ± 6.41	13.90 ± 2.93	15.20 ± 2.82	12.52 ± 4.74
Neutrophils, %	43.40 ± 3.13	40.40 ± 8.99	42.80 ± 8.93	43.60 ± 12.40
Lymphocytes, %	53.80 ± 4.21	55.60 ± 8.20	55.20 ± 8.64	54.00 ± 12.37
Monocytes, %	0.60 ± 0.55	1.00 ± 0.71	0.40 ± 0.55	1.00 ± 1.41
Total protein, g/dL	8.52 ± 0.48	8.32 ± 0.50	8.20 ± 0.86	8.12 ± 0.33
AST, U/L	79.80 ± 15.93	87.00 ± 13.19	81.60 ± 7.40	72.60 ± 11.01

Values are presented as mean ± SD of individual-animal measurements obtained on Day 0 before the initiation of dietary supplementation. Each treatment comprised five goats. CON, MOS2.5, MOS5, and MOS10 denote the groups assigned to receive 0, 2.5, 5.0, and 10.0 g of PKM-derived MOS-rich hydrolysate/goat/day, respectively. SD, standard deviation; RBC, red blood cell count; MCV, mean corpuscular volume; MCH, mean corpuscular hemoglobin; MCHC, mean corpuscular hemoglobin concentration; WBC, white blood cell count; AST, aspartate aminotransferase.

Table S4. Hematological indices measured before and 4 h after feeding in growing goats supplemented with PKM-derived MOS-rich hydrolysate.

Parameter	Sampling time	CON	MOS2.5	MOS5	MOS10	SEM	Linear *p*	Quadratic *p*
RBC, $\times 10^6/\mu L$	Before feeding (0 h)	14.28	12.72	13.84	14.68	0.28	0.150	<0.001
	4 h after feeding	14.04	13.84	15.04	14.40	0.32	0.190	0.560
	Overall mean	14.16	13.28	14.44	14.52	0.24	0.100	0.100
MCV, fL	Before feeding (0 h)	21.74	23.29	19.84	20.12	0.27	<0.001	0.260
	4 h after feeding	22.03	23.68	19.54	20.18	0.26	<0.001	0.440
	Overall mean	21.89	23.48	19.69	20.15	0.15	<0.001	0.310
MCH, pg	Before feeding (0 h)	7.54	8.63	7.71	7.33	0.13	0.080	<0.001
	4 h after feeding	8.14	8.50	7.50	7.65	0.12	0.010	0.550
	Overall mean	7.84	8.57	7.60	7.49	0.07	0.010	0.010
MCHC, g/dL	Before feeding (0 h)	35.66	35.87	36.79	35.40	0.34	0.930	0.050
	4 h after feeding	36.19	36.29	37.62	36.62	0.33	0.140	0.160
	Overall mean	35.92	36.08	37.21	36.01	0.22	0.290	0.030
WBC, $\times 10^3/\mu L$	Before feeding (0 h)	15.59	18.17	13.56	15.28	0.64	0.220	0.660
	4 h after feeding	15.54	16.26	13.58	15.89	0.78	0.690	0.380
	Overall mean	15.56	17.22	13.57	15.59	0.39	0.270	0.800
Neutrophils, %	Before feeding (0 h)	34.52	29.61	30.26	39.28	1.37	0.020	<0.001
	4 h after feeding	59.40	48.38	52.33	49.02	1.75	0.010	0.080
	Overall mean	46.96	38.99	41.29	44.15	0.93	0.210	<0.001
Lymphocytes, %	Before feeding (0 h)	65.85	56.33	61.87	53.98	1.61	0.010	0.720
	4 h after feeding	33.55	46.49	41.29	39.28	2.48	0.330	0.010
	Overall mean	49.70	51.41	51.58	46.63	1.37	0.150	0.020
Monocytes, %	Before feeding (0 h)	1.63	1.62	1.16	0.70	0.26	0.010	0.390
	4 h after feeding	3.39	3.48	3.31	3.55	0.28	0.800	0.780
	Overall mean	2.51	2.55	2.23	2.13	0.19	0.090	0.700

Values are least-squares means (n = 5 goats/treatment). CON, MOS2.5, MOS5, and MOS10 supplied 0, 2.5, 5.0, and 10.0 g of PKM-derived MOS-rich hydrolysate/goat/day, respectively. SEM, standard error of the mean; RBC, red blood cell count; MCV, mean corpuscular volume; MCH, mean corpuscular hemoglobin; MCHC, mean corpuscular hemoglobin concentration; WBC, white blood cell count. Linear and quadratic contrasts were constructed using the actual supplementation levels. Statistical significance was defined as $p < 0.05$, and a tendency was defined as $0.05 \leq p < 0.10$.

Table S5. Serum biochemical indices measured before and 4 h after feeding in growing goats supplemented with PKM-derived MOS-rich hydrolysate.

Parameter	Sampling time	CON	MOS2.5	MOS5	MOS10	SEM	Linear *p*	Quadratic *p*
Total protein, g/dL	Before feeding (0 h)	5.96	6.37	6.21	6.31	0.40	0.630	0.620
	4 h after feeding	6.08	6.05	6.27	6.23	0.17	0.690	0.730
	Overall mean	6.02	6.21	6.24	6.27	0.26	0.640	0.760
AST, U/L	Before feeding (0 h)	104.18	87.72	102.91	107.36	6.58	0.450	0.350
	4 h after feeding	111.94	93.15	115.08	108.02	4.86	0.730	0.200
	Overall mean	108.06	90.44	109.00	107.69	5.34	0.550	0.260
ALT, U/L	Before feeding (0 h)	26.52	24.82	21.08	22.44	2.35	0.310	0.740
	4 h after feeding	27.20	22.10	21.25	23.80	1.34	0.350	0.180
	Overall mean	26.86	23.46	21.17	23.12	1.59	0.290	0.390
ALP, U/L	Before feeding (0 h)	405.96	297.84	233.92	327.64	50.00	0.160	0.650
	4 h after feeding	433.82	356.32	262.14	353.26	19.00	0.070	0.420
	Overall mean	419.89	327.08	248.03	340.45	34.00	0.120	0.520

Values are least-squares means (n = 5 goats/treatment). CON, MOS2.5, MOS5, and MOS10 supplied 0, 2.5, 5.0, and 10.0 g of PKM-derived MOS-rich hydrolysate/goat/day, respectively. SEM, standard error of the mean; AST, aspartate aminotransferase; ALT, alanine aminotransferase; ALP, alkaline phosphatase. Linear and quadratic contrasts were constructed using the actual supplementation levels. Statistical significance was defined as $p < 0.05$, and a tendency was defined as $0.05 \le p < 0.10$.
